# Clade-wide fungal proteome analysis reveals structure–function conservation in divergent Dicer proteins

**DOI:** 10.1016/j.csbj.2025.11.009

**Published:** 2025-11-06

**Authors:** Lorena Melet, Jonathan Canan, Pablo Villalobos, Boris Vidal-Veuthey, Fabián González-Toro, Ivana Orellana, J. Andrés Rivas-Pardo, Juan P. Cárdenas, Carol Moraga, Víctor Castro-Fernández, Nathan R. Johnson, Elena A. Vidal

**Affiliations:** a Centro de Genómica y Bioinformática, Universidad Mayor, Santiago 8580745, Chile; b Agencia Nacional de Investigación y Desarrollo-Millennium Science Initiative Program, Millennium Institute for Integrative Biology (iBio), Santiago 7500565, Chile; c Agencia Nacional de Investigación y Desarrollo-Millennium Science Initiative Program, Millennium Nucleus in Data Science for Plant Resilience (Phytolearning), Santiago 8370186, Chile; d Departamento de Biología, Facultad de Ciencias, Universidad de Chile, Santiago 7800003, Chile; e Instituto de Ciencias de la Ingeniería, Universidad de O′Higgins, Rancagua 2841959, Chile; f Centro UOH de Bioingeniería (CUBI), Universidad de O’Higgins, Rancagua 2841959, Chile

**Keywords:** Fungi, Dicer, PAZ, RNAi

## Abstract

Dicers (Dcrs) are central proteins involved in the biogenesis of small RNAs (sRNAs). Most of the knowledge on Dcr structure, function and evolution comes from studies in animals and plants. Comparatively, less is known in fungi, a genetically and ecologically diverse group with important roles in ecosystems, agriculture, medicine, and biotechnology. While canonical Dcrs contain a well-defined domain architecture, most fungal Dcrs lack one or more identifiable canonical domains, raising questions about how RNA-binding and sRNA-processing is retained. We conducted an extensive survey of fungal Dcrs, analyzing 1592 proteomes across nine phyla. We found a diversity of domain architectures, with some lacking identifiable Piwi, Argonaute and Zwille (PAZ), Helicase, and/or double-stranded RNA-binding (dsRBD) domains. Phylogenetic analyses showed that different Dcr classes are distributed across distinct clades that often align with fungal taxonomic groups. Despite the lack of canonical domain architectures, fungal Dcrs fold into characteristic structures and show PAZ-like folds, displaying an OB-fold core typical of PAZ domains. Molecular simulation analyses further indicate that these divergent Dcrs maintain key RNA-binding surfaces for proper sRNA processing. Our results indicate a remarkable evolutionary plasticity of Dcr in fungi, showing that essential sRNA processing functions can be retained through structural conservation, and highlighting fungi as models to study the modular evolution of the RNAi machinery in eukaryotes.

## Graphical Abstract

**Figure:**
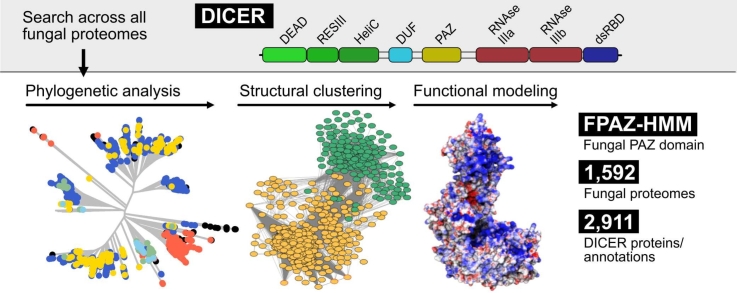

## 1 Introduction

RNA interference (RNAi) is a conserved eukaryotic mechanism with diverse roles, including gene expression regulation [1], antiviral defense [2], genome stability [3], and communication between cells and across species [4], [5], [6]. RNAi is mediated by small RNA (sRNA) molecules broadly categorized as 18–30 nucleotides in length, which are generated through the cleavage of exogenous or endogenous double-stranded RNA (dsRNA) precursors. These sRNAs are loaded into RNA-induced silencing complexes, which use sequence complementarity to target nucleic acid sequences for transcriptional or post-transcriptional gene silencing [7], [8]. Among the proteins responsible for processing sRNAs from dsRNAs, Dicer (Dcr) plays a central role, particularly in the small interfering RNA (siRNA) and microRNA (miRNA) pathways.

Numerous studies have elucidated the functions of Dcr domains in recognizing and processing their canonical substrates, including double-stranded RNAs and single-stranded hairpin precursors of miRNAs (pre-miRNAs). Structurally, Dcr proteins exhibit an L-shaped, modular, multidomain architecture that can be broadly divided into three regions along their three-dimensional organization: a base, a core (body), and a head. These regions reflect both domain composition and spatial-functional arrangement [9], [10], [11]. The base (N-terminal region) contains the helicase domain, comprised of three subdomains: DExD/H-box, RESIII, and helicase C-terminal domains. These serve as the entry point for dsRNA substrates, recognizing and binding them before unwinding the RNA duplex to ensure proper positioning. Evidence shows that in some species and Dcr variants the helicase domain may or may not be involved in ATP hydrolysis, a function that has been related to recognition and processing of blunt-ended dsRNAs such as viral RNAs or endogenous dsRNAs [12], [13], [14]. The core (central region) includes the domain of unknown function (DUF283) and dsRBD. DUF283 functions primarily as a protein–protein interaction module, selectively interacting with dsRBD-containing cofactors such as DRB4 and HYL1 in Arabidopsis [15], [16], or proteins such as ADAR1 in mammals [17]. The dsRBD enhances the stability and specificity of enzyme-substrate interaction contributing to the fidelity of the cleavage reaction [18]. At the catalytic core, the tandem RNase IIIa and IIIb domains form a dimer that cleaves both strands of the dsRNA duplex. The head (C-terminal region) contains the Piwi, Argonaute and Zwille (PAZ) domain, which binds the 3’ overhangs of dsRNA substrates, acting as an anchoring point that guides the cleavage site [19]. In the three-dimensional structure of Dcr, the PAZ domain is connected to the RNase III catalytic center by a connector α-helix. This helix acts as a molecular ruler, setting the distance between the RNA-binding PAZ domain and the RNase III domains to define the length of the sRNAs produced (∼21–25 nt) [20].

While this canonical domain architecture is conserved across most of the Dcr proteins in animals and plants, reduced Dcr proteins have also been described. The minimal Dcr protein from the protist *Giardia intestinalis* consists solely of the tandem RNase III domains connected through a connector helix to a platform region, yet remains fully competent in cleaving dsRNA substrates [20]. Interestingly, many Dcr proteins in fungi have been reported to lack identifiable Helicase, PAZ and/or dsRBD domains, suggesting a widespread presence of non-canonical Dcrs [21], [22], [23]. This diversity positions fungal Dcrs as a valuable model for exploring how ancestral RNase III enzymes evolved into the complex, multidomain Dcr proteins found in higher eukaryotes.

Fungi comprise a large and diverse group of eukaryotic organisms, currently classified into at least 12 major phyla [24], with some recent taxonomic studies recognizing up to 18 or 19 distinct phyla based on phylogenomic evidence [25], [26], [27]. Most described fungal species to date belong to the higher fungi Ascomycota and Basidiomycota, which together form the clade Dikarya. Other fungal phyla (e.g., Mucoromycota, Chytridiomycota, Zoopagomycota) represent a smaller portion of known fungal diversity, though they are ecologically important and likely under-sampled [25].

In fungi, RNAi plays a crucial role not only in genome defense but also in the regulation of development, pathogenicity, and inter-species interactions [28], [29]. Fungal RNAi pathways have been implicated in transposon silencing, antiviral protection, and the regulation of sexual and vegetative processes, reflecting their functional versatility across diverse lineages [30], [31]. Moreover, RNAi contributes to fungal–plant communication through the secretion of sRNAs that can modulate host gene expression, a phenomenon described in both pathogenic and mutualistic species [6], [32]. Despite this functional diversity, the molecular components and evolutionary trajectories of fungal RNAi remain far less characterized than in animals or plants [22], [33].

Multiple approaches have been used to identify RNAi-related proteins in fungi, ranging from focused studies in budding yeasts such as *S. castelli*, *K. polysporus*, and *C. albicans*, in selected Basidiomycota species within the Agaricomycotina, or in microsporidian parasites from different animal hosts [22], [23], [34], to large-scale comparative surveys such as the FunRNA database, which catalogued RNAi components across 131 fungal genomes from the Ascomycota, Basidiomycota, Chytridiomycota, Zygomycota and Microsporidia [21]. These studies have shown that the number and architecture of Dcr proteins vary significantly across fungal species. While many fungi encode two Dcr paralogs [35], [36], these can differ in sequence and domain organization, which may reflect functional specialization [22], [37]. However, there are also fungal lineages where only a single Dcr has been identified [22], [38] and others, including members of the Saccharomycotina and some Basidiomycota, where Dcr genes appear to be absent altogether [21], [22], [23]. Notably, the apparent absence of Dcr domains, such as PAZ or dsRBD in some fungal proteins may result from limitations in domain annotation pipelines rather than true biological loss. For example, domains like PAZ may be highly divergent in fungi, and thus escape identification by current probabilistic models based on protein alignments of animal and plant-derived PAZ domains [39]. However, albeit divergent, PAZ-like folds have been reported in fungal Dcrs such as Dicer from *S. pombe* [40], hinting at tridimensional structure conservation, and at the importance of incorporating structural analyses to generate hypotheses on fungal Dcr function.

The number of publicly available annotated and reference fungal genomes in the NCBI genome database has expanded more than eightfold over the past decade, from 234 genomes in 2015 to nearly 1900 by 2025, presenting a prime opportunity to study Dcr diversity at a wide phylogenetic scale. Furthermore, integration of structure prediction methods like AlphaFold [41] can help to determine whether structural conservation is widespread across fungal Dcrs despite apparent sequence diversity.

In this study, we present a comprehensive analysis of Dcr proteins across fungi, revealing widespread non-canonical architectures that often lack identifiable PAZ, Helicase, or dsRBD domains. Through a combination of phylogenetic analyses, structural modeling, and molecular docking predictions, our results suggest that many of these divergent Dcrs may retain key features for RNA processing, including structurally conserved PAZ-like regions. Our findings expand the understanding of Dcr diversity in fungi and suggest that RNAi functionality can be maintained despite extensive domain loss or divergence.

## 2 Results

### 2.1 Comprehensive identification of Dcr proteins in fungi

**Fig. 1:**
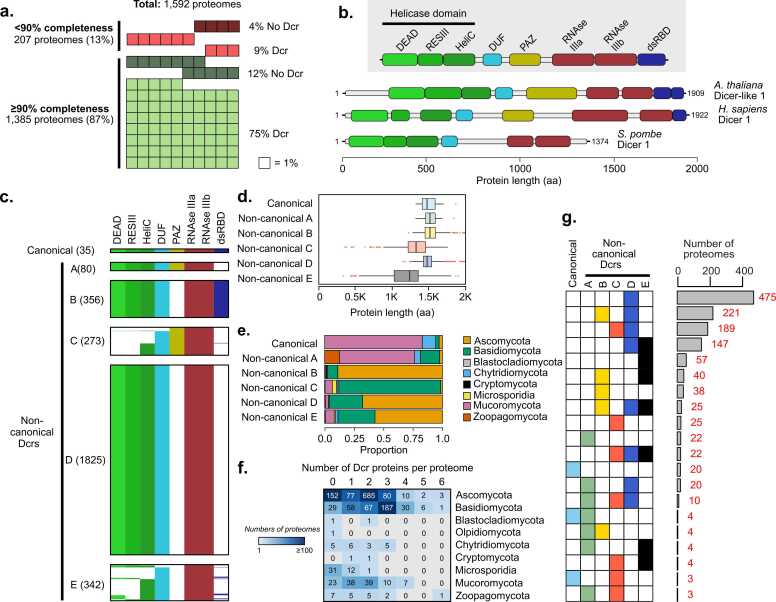
Domain-based classification and distribution of Dicer proteins across fungal phyla. (**a**) Summary of the 1592 annotated fungal proteomes analyzed for the identification of Dcr proteins, showing the percentage of < 90 % and ≥ 90 % BUSCO completeness proteomes. No Dcr: no Dcr detected by HMMER analysis; Dcr: Dcr detected by HMMER analysis (**b**) Representative domain architectures of known Dcr proteins from *Arabidopsis thaliana*, *Homo sapiens*, and *Schizosaccharomyces pombe*, illustrating the conserved domain composition: DEAD, ResIII, Helicase C (HeliC), DUF283, PAZ, RNase IIIa and IIIb, and dsRNA-binding domain (dsRBD). Protein lengths are shown in amino acids (aa). (**c**) Classifi**c**ation of all predicted fungal Dcr proteins into six categories based on the presence or absence of key domains. Canonical Dcrs retain a full complement of conserved domains, while non-canonical groups exhibit partial or divergent architectures. Each file represents a Dcr protein, and the colors represent whether a domain was detected by HMMER (green shades: Helicase subdomains; bright cyan: DUF283 domain; olive yellow: PAZ domain; brick red: RNaseIII domain; indigo blue: dsRBD domain). White color indicates that the specific domain was not detected by HMMER. (**d**) Average protein length (in aa) per Dcr category. (**e**) Distribution of Dcr categories across fungal phyla, highlighting differences in domain conservation and structural diversity among lineages. (**f**) Variability in Dcr family expansion across phyla. Numbers in the boxes represent the number of proteomes per phyla containing 0–6 Dcr proteins. (**g**) Combinations of Dcr categories present in fungal proteomes. Each row shows the presence (colored square) or absence (white square) of canonical and non-canonical Dcrs (A–E) in the proteome. Bars on the right represent the number of proteomes presenting a given combination of Dcr categories (total number in red).

To comprehensively catalog fungal Dcr proteins, we retrieved all available fungal reference proteomes from the NCBI, obtaining 1592 datasets representing 1418 unique species from nine Phyla (Supplementary Table 1). These included 910 species of Ascomycota (64.2 % of the total unique species), 333 of Basidiomycota (23.5 % of the total unique species), and 175 non-Dikarya species, including 95 Mucoromycota (6.7 % of the total unique species), 20 Zoopagomycota (1.4 % of the total unique species), 19 Chytridiomycota (1.3 % of the total unique species), 36 Microsporidia (2.5 % of the total unique species), 2 Cryptomycota (0.1 % of the total unique species), 2 Blastocladiomycota (0.1 % of the total unique species), and 1 Olpidiomycota (0.1 % of the total unique species). This dataset represents a substantial expansion compared with the FunRNA database, which included only 95 unique species of Ascomycota, 31 of Basidiomycota and 10 of non-Dikarya species from Mucoromycota, Chytridiomycota and Blastocladiomycota. The selected proteomes were predicted from genomes labeled as 'reference' or 'annotated' in NCBI, excluding those classified as atypical. These genomes meet the criteria for reference-quality assemblies. We evaluated the completeness of each proteome using BUSCO [42], choosing a completeness of ≥ 90 % as a practical threshold to distinguish proteomes with high completeness. As expected, most of the reference proteomes exhibited ≥ 90 % completeness on either the fungi-wide or phylum-specific datasets (Fig. 1A, Supplementary Table 1). This dataset expands the taxonomic scope for the identification and comparative analysis of fungal Dcr proteins, as compared with previous studies [21], [23], [34].

To identify putative Dcr proteins, we screened the proteomes for canonical Dcr-associated domains previously described in plants and animals. Domain searches were performed using HMMER, following methodologies established for Dcr identification in fungi [21] and other organisms [43]. A protein was classified as a putative Dcr if it met two criteria: 1. the presence of two, tandem RNase III domains, essential for dsRNA cleavage, and 2. at least one RNA-binding domain, including Helicase subdomains DExD/H-box, ResIII or HeliC, DUF283, PAZ, or dsRBD. This approach identified 2911 putative Dcr proteins across 1346 proteomes (84.4 % of the total analyzed proteomes), representing 1203 unique species from eight fungal phyla (Supplementary Table 2, Supplementary Table 3). To validate these predictions, we used an independent domain annotation approach using InterProScan, which showed 100 % concordance with the HMMER-based results (Supplementary Table 2).

Interestingly, we found no evidence of Dcr proteins in 249 proteomes from 231 unique species. From these, 186 proteomes were ≥ 90 % BUSCO completeness (12 % of the total proteomes) and 63 proteomes were < 90 % BUSCO completeness (4 % of the total proteomes) (Fig. 1A, Supplementary Table 4). The lack of Dcrs in a fraction of proteomes with ≥ 90 % completeness suggests that these likely represent true gene losses rather than artifacts related to incomplete assemblies or gene annotation. However, in proteomes with < 90 % completeness, the absence of Dcrs should be interpreted with caution, as it may be related to incomplete or fragmented gene models rather than true gene loss events. Among the proteomes with ≥ 90 % completeness and no detectable Dcrs we identified 145 Ascomycota species, of which 113 belong to the subphylum Saccharomycotina, including members from the classes Dipodascomycetes, Saccharomycetes, Pichiomycetes and Trigonopsidomycetes (Supplementary Table 4). This subphylum encompasses most Ascomycete yeasts, several of which have previously been reported to lack a functional RNAi machinery [22]. In addition, species lacking Dcrs were also found in Basidiomycota, Olpidiomycota, Blastocladiomycota, Chytridiomycota, Microsporidia, Mucoromycota, and Zoopagomycota (Supplementary Table 4), suggesting that Dcr protein loss has occurred multiple times independently across fungal lineages.

### 2.2 Widespread domain architecture variation and predominance of non-canonical Dcr types in fungi

To characterize the domain composition of fungal Dcrs, we referred to the canonical domains typically found in Dcr proteins (Fig. 1B). Using these domain features as a guide, we classified the 2911 identified Dcr proteins into six categories based on the presence or absence of domains detected through HMMER analysis (Fig. 1C, Supplementary Table 2). Analysis of the types of domains found across the fungal dataset revealed the predominance of non-canonical forms and the high frequency of Dcr proteins where the PAZ and/or dsRBD domains could not be detected. Proteins containing all expected domains were classified as canonical Dcrs, representing a small subset of the dataset (35 proteins, 1.2 % of the total identified Dcr proteins). Proteins with one or more missing domains were designated as non-canonical Dcrs and further grouped according to the specific patterns of domain loss. Non-canonical A includes proteins lacking only the dsRBD domain (80 proteins, 2.7 % of the total identified Dcr proteins), while non-canonical B consists of those missing the PAZ domain but retaining all other domains (356 proteins, 12.2 % of the total identified Dcr proteins). Non-canonical C comprises proteins in which Helicase subdomains and dsRBD are absent (273 proteins, 9.4 % of the total identified Dcr proteins). The most abundant group, non-canonical D, consists of proteins lacking both the PAZ and dsRBD domains (1825 proteins, 62.7 % of the total identified Dcr proteins). Finally, non-canonical E includes proteins in which three or more domains could not be detected (342 proteins, 11.8 % of the total identified Dcr proteins).

The PAZ domain, central to RNA binding and precise determination of sRNA length, was undetectable in the vast majority of Dcr proteins, with 86.7 % lacking a recognizable PAZ domain. However, protein length analysis revealed that proteins without a detectable PAZ, particularly those in the non-canonical B and D categories, are comparable in size to canonical and non-canonical A proteins that do contain this domain (Fig. 1D). This suggests that the PAZ domain may still be present but has undergone substantial sequence divergence relative to the canonical PAZ (cPAZ) described in plants and animals, rendering it undetectable by standard HMM-based methods. Consistent with this idea, the current PAZ HMM matrix used in this analysis (PF02170/IPR3100) was constructed from Dcrs and ARGONAUTE proteins mainly from plants and animals, with negligible representation of fungal Dcrs. Similarly, the dsRBD domain, also involved in RNA binding, was frequently undetected, with 84.8 % of proteins missing this domain.

Dcrs in categories non-canonical C and E generally exhibited smaller sized proteins than other groups (Fig. 1D). Associating this with the lack of detection of terminal domains discovered in many of these proteins (Fig. 1C), we reasoned that this might be due to truncation. To explore this, we measured the N-terminal and C-terminal lengths (Supplementary Fig. 1) to the nearest highly conserved domain (DUF283 and RN*Ase*IIIb, respectively). This pointed to a shorter C-terminus in non-canonical E proteins, further suggesting truncation in this category (Supplementary Fig. S1A). Notably, other categories with no detectable dsRBD (non-canonical A, C, and D) did not display this truncation, possibly indicating a divergent dsRBD domain is present. Exploring the N-terminus showed shorter proteins for both non-canonical C and E proteins, both categories where at least one helicase subdomain is not detected (Supplementary Fig. S1B). This suggests that this may be a true truncation and partial or total loss of the helicase domain and may explain the reduced size of these proteins in general.

The distribution of Dcr categories varies substantially among fungal phyla (Fig. 1E). Canonical Dcrs and non-canonical A proteins, both of which retain a cPAZ domain, are enriched in Mucoromycota, a phylum composed of mychorrizal symbionts, plant and animal pathogens, fungal parasites and decomposers of organic matter [44]. In contrast, non-canonical C proteins, which also retain a cPAZ domain, were primarily found in Basidiomycota. Non-canonical B, D, and E proteins, lacking a cPAZ domain, are most prevalent in Ascomycota, with some also found in Basidiomycota. The number of dcr genes per genome, based on our annotation pipeline using HMMER, also varied by phylum; most Ascomycota proteomes have two dcr genes (685 proteomes, 68 % of Ascomycota proteomes) while Basidiomycota more frequently possess three (187 proteomes, 49.5 % of Basidiomycota proteomes). Strikingly, Microsporidia, a group of obligate intracellular parasites, either lacked dcrs entirely (31 proteomes, 70.5 % of Microsporidia proteomes) or contained only a single dcr (12 proteomes, 27.3 % of Microsporidia proteomes) (Fig. 1F), consistent with previously described reductions in gene contents in this lineage [45].

We also analyzed the distribution of Dcr category combinations across fungal species (Fig. 1G). Most species contain non-canonical D Dcrs as the sole Dcr type (475 species), or in combination with non-canonical B (221 species), C (189 species), or E (147 species). Interestingly, canonical Dcrs were either found alone or in combination with non-canonical A or C, both of which retain a cPAZ domain. These patterns underscore the diversity in both the number and types of Dcr proteins across fungal taxa, suggesting lineage-specific adaptations in the RNAi machinery, likely in response to differing ecological and/or evolutionary pressures.

### 2.3 Clade-specific distribution of canonical and non-canonical Dcrs suggests independent diversification of RNAi pathways in fungi

**Fig. 2:**
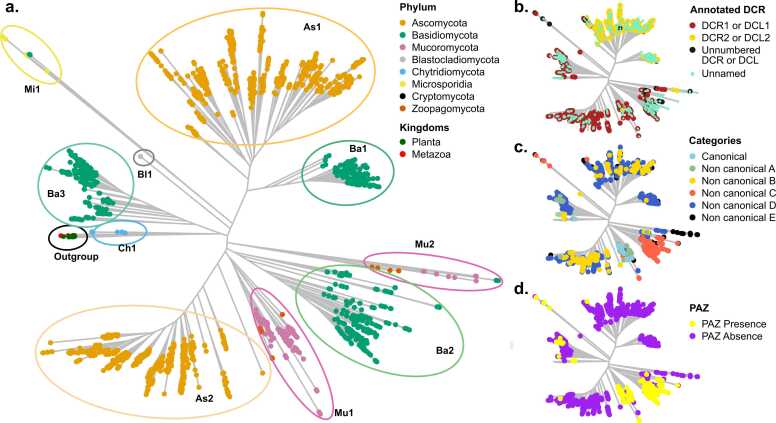
Phylogenetic and functional classification of fungal Dcr proteins. (**a**) Maximum-likelihood phylogenetic tree showing the distribution of Dcr proteins across fungal phyla, as well as representative outgroups from plants and metazoans. Colored ovals highlight major clades identified within fungal lineages, labeled as As1–As2 (Ascomycota), Ba1–Ba3 (Basidiomycota), Mu1–Mu2 (Mucoromycota and Zoopagomycota), Bl1 (Blastocladiomycota) or Ch1 (Chytridiomycota), based on their phylogenetic position. (**b**) Naming of Dcr proteins according to UniProtKB, distinguishing Dcr1/Dcl1 (Dark red), Dcr2/Dcl2 (yellow), unnumbered Dcr/Dcl entries (black), and proteins with no assigned name (unnamed, light green). (**c**) Classification of fungal Dcrs into canonical and non-canonical categories (A–E), based on domain composition. (**d**) Mapping of canonical PAZ domain presence (yellow) or absence (purple) across the phylogeny, according to HMMER detection.

To investigate the phylogenetic relationships of fungal Dcr proteins, we constructed a sequence similarity network, considering a cut-off of 80 % coverage and 50 % identity between connected nodes. 2636 Dcr proteins met these criteria and were clustered into 149 groups (Supplementary Table 2). Multiple sequence alignments (MSAs) were generated for each cluster and integrated into a global alignment, which was used to construct a maximum-likelihood phylogenetic tree, including outgroup sequences from plants and metazoans (Fig. 2A). The analysis revealed a set of well-supported clades that we defined as major Dcr groups (Supplementary Files 1–10). These groups correspond to fungal taxonomic lineages, with Ascomycota sequences separating into two major clades (As1 and As2) and Basidiomycota sequences into three distinct clades (Ba1, Ba2, Ba3). In contrast, sequences from non-Dikarya fungi formed more scattered groups, forming independent clades (Mu1, Mu2, Mi1, Bl1, Ch1), suggesting that Dcr diversification in non-Dikarya followed an independent evolutionary trajectory. The outgroup sequences formed a distinct branch, supporting the overall topology and robustness of the tree (Fig. 2A). To further evaluate the reliability of the alignments supporting these clades, we evaluated alignment completeness for each major clade using AliStat [86] (Supplementary Table 5). We found that the global conservation scores (C_a_) for the major clades, Ascomycota and Basidiomycota, were generally low, indicating they substantial sequence variation likely associated with the deep evolutionary age of these groups. However, their higher maximum conservation (C_r_) and pairwise conservation (C_ij_) values provide confidence in the quality and alignment accuracy of the MSA. Moreover, regions of strong conservation were detected, as reflected by a maximum column conservation rate (C_c_) of 1.0 across all clades. Visual inspection of the alignment revealed well-defined conserved blocks primarily corresponding to the RN*Ase*III and DUF283 domains, supporting the structural coherence of these elements despite the extensive overall sequence divergence.

To evaluate whether clade assignments relate to previously reported Dcr annotations, we mapped proteins annotated as Dcrs in UniProtKB onto the phylogenetic tree (Fig. 2B). These include proteins labeled as Dcr1, Dcr2, Dcr-like1 (Dcl1), Dcr-like2 (Dcl2), as well as others annotated more generically as “Dicer” or “Dicer-like”. We found that proteins annotated as Dcr1 or Dcl1 are predominantly located within the Ascomycota As2 clade and the Basidiomycota clades Ba2 and Ba3. Dcr1/Dcl1 proteins are also observed in the Mu1 and Mu2 clades, as well as some identified as Dcr2/Dcl2 in Mu2. These ancient duplications forming Ba2/3 and Mu1/2 point to an unclear resolution of the ancestral proteins and possible paraphyletic naming. However, these are the exceptions. In general, Dcrs with similar annotations tend to group within the same clades, suggesting that existing naming conventions reflect underlying evolutionary relationships, even if their functional roles have not been fully confirmed. Notably, most of the Dcrs identified in our analysis remain unannotated in public databases (Fig. 2B, Supplementary Table 2), underscoring the extensive under-representation of fungal Dcr diversity and abundance.

To further explore the relationship between phylogeny and domain architecture, we mapped canonical and non-canonical classifications onto the phylogenetic tree. Canonical Dcrs clustered primarily within the Mu1 clade, and to a lesser extent, in the Mu2 clade, consistent with their enrichment in the Mucoromycota phylum (Fig. 1E, Fig. 2C). Non-canonical A and C proteins, which, as canonical Dcrs, retain a cPAZ domain (Fig. 1C, Fig. 2D), also show a restricted distribution, with non-canonical C primarily associated with the Ba2 clade and non-canonical A with Ba3. In contrast, non-canonical B, D and E proteins, which lack a cPAZ domain (Fig. 2D), were widely distributed across the tree (Fig. 2C). The restricted phylogenetic distribution of cPAZ-containing Dcrs suggests this domain is not broadly conserved across fungal lineages; rather, its retention appears to be lineage-specific and may reflect convergent retention or loss events in different evolutionary contexts.

The helicase subdomains DExD/H-box, ResIII, and HeliC also showed differential representation across fungal clades, although most Dcrs retained Helicases with all three subdomains (Fig. 1C, Supplementary Fig. 2 A). Notably, Dcrs lacking all helicase components, or retaining only the HeliC subdomain, were mainly found in the Ba2 clade, suggesting that Basidiomycota harbor both helicase-complete Dcrs (e.g., in Ba1 and Ba3) and more divergent forms potentially lacking ATPase activity (Ba2). A similar pattern was observed in Mucoromycota, with Mu1 Dcrs containing complete helicases and Mu2 Dcrs showing partial loss of subdomains. Additional incomplete helicase architectures were also found in the Microsporidian clade Mi1. In contrast, the dsRBD domain, another typical component of canonical Dcrs, but rarely detected in our analysis, was present in proteins found in diverse clades across the tree (Supplementary Fig. 2B). These results underscore the evolutionary plasticity of fungal Dcrs, suggesting that distinct selective pressures have driven domain loss, retention, or divergence across fungal lineages.

### 2.4 Fungal Dcrs have conserved structural features despite sequence divergence

**Fig. 3:**
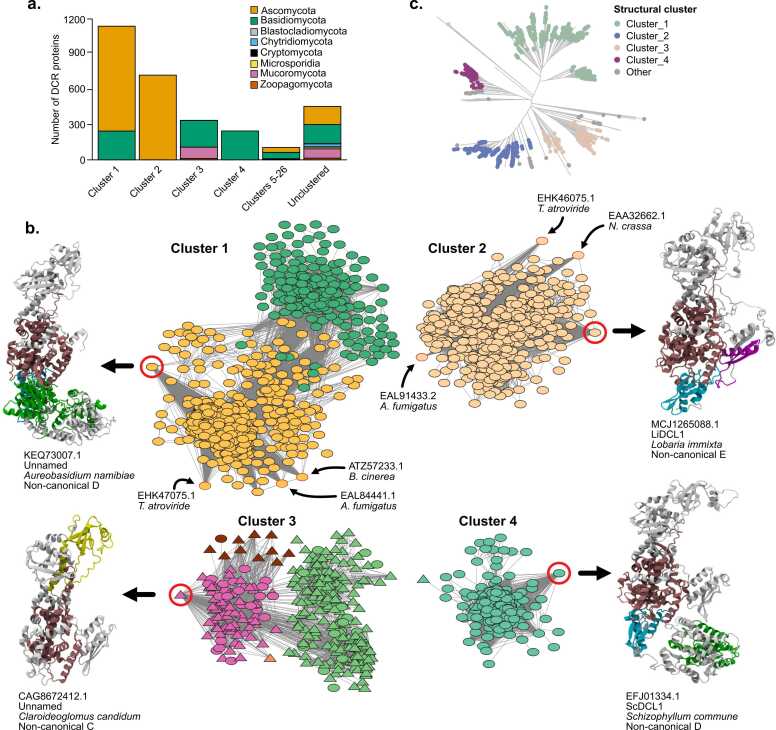
Structural similarity network analysis reveals strong structural conservation of Dcr proteins. (a) Taxonomic composition of each structural cluster, shown as the number of Dcr proteins per fungal phylum. (**b**) Structural similarity network of predicted Dcr proteins, clustered based on TM-score ≥ 0.9. Each node represents a protein structure; edges indicate pairwise structural similarity (only edges of TM-score 0.9 or more are shown). Node colors indicate the clade classification defined in Fig. 2A (e.g., As1, As2, Ba1–Ba3, Mu1–Mu2), allowing direct comparison between structural and phylogenetic groupings. The most connected node (hub) from selected modules is shown with a red circle and its corresponding AlphaFold-predicted 3D structure is represented on the side, including protein code, Dicer naming according to UniProtKB (unnamed: no name available), the species name and Dcr group (according to Fig. 1 classification). Proteins displayed correspond to representative members of non-canonical Dcr groups B, C, and E. Structural domains are colored as follows: PAZ (yellow), Helicase (green), RNase III (dark red/burgundy), DUF283 (cyan), and dsRBD (purple). Regions without domain predictions are shown in grey. Triangular nodes correspond to Dcr proteins with a canonical PAZ domain (cPAZ); oval nodes indicate Dcrs with non-canonical PAZ domains. **(c**) Phylogenetic tree of all Dcr proteins, with tips colored according to structural cluster membership, illustrating the correspondence between structural similarity and evolutionary lineage. “Other” corresponds to clusters 5–26.

The sequence similarity network and phylogenetic analyses suggest a high degree of sequence divergence among fungal Dcr proteins, with several instances in which key domains typically associated with Dcr function were not detected, likely due to either their absence or low sequence conservation. To explore whether these proteins might nonetheless retain conserved three-dimensional features, we generated structural predictions for all Dcr proteins using AlphaFold [41]. Protein models displaying a predicted Local Distance Difference Test (pLDDT) score greater than or equal to 70 were selected, and a structural similarity network was constructed, considering a template modeling (TM) score of ≥ 0.9 between connected nodes as a threshold. Among the selected models, the average pLDDT score was 75, supporting the overall reliability of the structural predictions. A total of 2475 proteins met this criterion and were grouped into 26 clusters (Supplementary Table 2). Strikingly, nearly 95 % of phylogenetically analyzed proteins are found within just four major structural clusters (Fig. 3A), suggesting core structural features are widely conserved. Each cluster showed a distinct representation of fungal phyla (Fig. 3A, B). Clusters 1 and 2 contain the majority of Dcr proteins, with cluster 1 including predominantly Ascomycota sequences and a smaller subset from Basidiomycota. This cluster harbors Dcrs with experimentally validated roles in sRNA processing such as *Botrytis cinerea* Dcr2 [6], [46], [32], *Aspergillus fumigatus* Dcr2 [47] or *Trichoderma atroviride* Dcr2 [48], [49], [50]. Cluster 2 is predominantly composed of Ascomycota sequences, and includes *A. fumigatus* Dcr1, *T. atroviride* Dcr1 and *Neurospora crassa* Dcr1 [51]. Clusters 3 and 4 include fewer proteins, mainly from Basidiomycota and Mucoromycota (cluster 3) or exclusively from Basidiomycota (cluster 4). The remaining smaller clusters consist of Ascomycota and Basidiomycota Dcrs and may represent functionally divergent paralogs present in these phyla (Fig. 3A, B).

The four major structural clades aligned well with the phylogenetic clades identified in our sequence-based analysis (Fig. 3C), indicating that despite extensive sequence divergence and domain variability, fungal Dcrs retain conserved structural architectures within evolutionary lineages. Cluster 1 contains sequences from clades As1 and Ba1, cluster 2 from As2, and cluster 3 from Ba2 and Mu1. Cluster 4 primarily contains Ba3, indicating that this is a unique lineage in Basidiomycota. Importantly, while proteins annotated as Dcr2 in UniProtKB majorly fall into Cluster 1, proteins annotated as Dcr1 are located into clusters 2, 3 and 4, indicating greater structural and likely functional diversity among Dcr1-labeled proteins. These findings underscore the need for systematic re-evaluation of Dcr annotations grounded in structural and evolutionary evidence rather than existing paralog labels.

The other cluster proteins (those falling outside the four major structural groups) may represent highly divergent Dcr variants, truncated sequences, or lineage-specific proteins with distinct structural features that deviate from the dominant structural folds. This group contains members from several phylogenetic clades, particularly dikarya fungi, and also includes Mucoromycota and Zoopagomycota Dcrs from clade Mu2 (Figs. 2B, 3C). It also includes all Dcrs from Blastocladiomycota clade Bl1, Microsporidia clade Mi1 and nearly all from Chytridiomycota clade Ch1, indicating substantial structural divergence in Dcr proteins in these non-Dikarya fungal lineages.

To further explore folding patterns, we examined the structure of the most connected (central) node within each major structural cluster (Fig. 3B). These representative proteins span different Dcr categories, with cluster 1 represented by a non-canonical D protein from *Aureobasidium namibiae* (clade As1), cluster 2 represented by *Li*Dcl1, a non-canonical E protein from *Lobaria immixta* (clade As2), cluster 3 represented by a non-canonical C protein from *Claroideoglomus candidum* (clade Mu1), and cluster 4 represented by *Sc*Dcl1, a non-canonical D protein from *Schizophyllum commune* (clade Ba3). Despite differences in domain composition detected by HMMER analysis, AlphaFold models of these proteins show broadly similar L-shaped Dcr architectures, comprising head, core and base regions. Importantly, the structural models revealed PAZ-like folds in the head region of representatives from clusters 1,2 and 4, even though cPAZ domains were not detectable at the sequence level in these proteins. This suggests that the RNA-binding functionality associated with PAZ might be structurally preserved in Dcrs despite substantial divergence, particularly in the more widely distributed non-canonical Dcr types.

### 2.5 Ancestral state reconstruction supports an early origin and selective retention of the cPAZ domain in Fungi

**Fig. 4:**
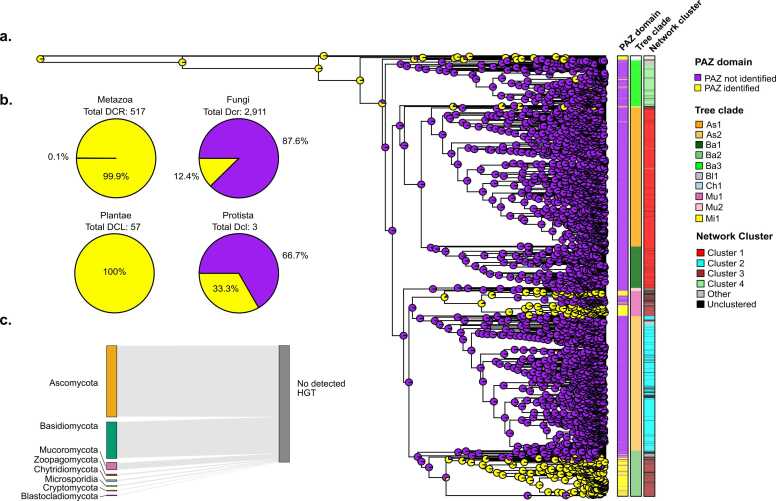
Evolutionary reconstruction of the PAZ domain in fungal Dcr proteins. (**a**) Phylogenetic tree of fungal Dcr proteins with ancestral state reconstruction for the canonical PAZ domain. Pie charts at internal nodes represent the inferred probability of PAZ domain presence (yellow) or absence (purple) using maximum likelihood estimation. Additional metadata layers include the presence/absence of a canonical PAZ domain, phylogenetic group according to Fig. 2(e.g., As1, Ba2, Mu1), and structural cluster as defined in Fig. 3. (**b**) Proportion of Dcr proteins with a canonical PAZ domain across eukaryotic groups Metazoa, Fungi, Plantae and Protista, highlighting variation in PAZ domain conservation. (**c**) Horizontal gene transfer (HGT) analysis. No recent inter-kingdom HGT events involving Dcr proteins were detected between fungi and plants, animals, or protists.

To gain deeper insights into the evolutionary history of the PAZ domain in fungi, we performed an ancestral character estimation (ACE) analysis to determine whether the cPAZ domain was present in the last common ancestor of fungal Dcrs (Fig. 4A). The ACE analysis was based on the presence or absence of the cPAZ domain, mapped onto the Dcr phylogeny. The reconstruction suggests that the common ancestor of fungal Dcrs most likely harbored a cPAZ domain, supporting the hypothesis that this domain is an ancestral feature of fungal Dcrs. Despite its ancestral origin, the phylogenetic distribution of cPAZ shows strong lineage-specific conservation patterns (Fig. 4A), consistent with those observed in the phylogenetic analysis (Fig. 2D) and structural clustering (Fig. 3A, C). The presence of the cPAZ domain in these clades may reflect convergent retention under shared selective pressures, or alternatively, could be the result of horizontal gene transfer (HGT) from lineages such as plants or animals, where the cPAZ domain is nearly ubiquitous. Indeed, nearly all Dcr proteins in metazoans (99.9 % of Dcrs) and plants (100 % of Dcrs) contain a cPAZ domain in contrast to only 12.4 % of fungal Dcrs (Fig. 4B). This stark difference likely reflects a greater evolutionary plasticity of the PAZ domain in fungi, where structural conservation can be retained despite extensive sequence divergence or domain reconfiguration. We evaluated the possibility of Dcr HGT from plants or animals to fungi, however our analysis did not find evidence supporting these events (Fig. 4C). No strong phylogenetic signals of HGT were detected between fungi and other major eukaryotic lineages. However, this outcome may reflect a methodological limitation of HGTector, which is optimized for detecting relatively recent transfer events and has reduced sensitivity for ancient or highly divergent ones. Therefore, our findings primarily support the absence of recent HGT involving fungal Dcrs, while the occurrence of ancient or deeply diverged transfer events cannot be completely ruled out.

These findings are consistent with a model in which the cPAZ may have been selectively retained in specific fungal lineages, while its apparent absence in most fungal Dcrs could instead reflect extensive sequence divergence rather than true domain loss. This interpretation is supported by the similar protein lengths observed between Dcrs with and without a cPAZ (Fig. 1D), as well as by structural predictions suggesting the presence of a putative conserved PAZ-like fold in proteins lacking a detectable cPAZ (Fig. 3C).

### 2.6 A sequence-divergent yet structurally conserved PAZ domain defines fungal Dcr diversity

**Fig. 5:**
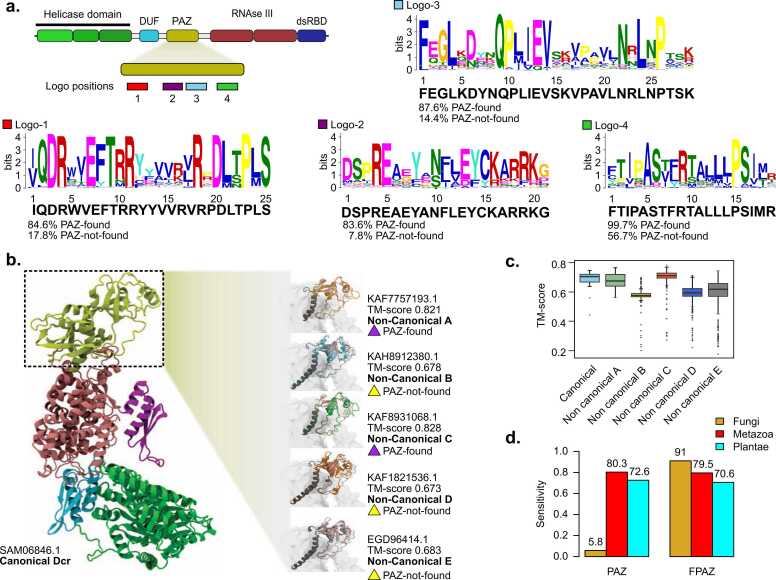
Motif conservation and structural variability of PAZ domains in fungal Dicer-like proteins. (a) Conserved sequence motifs identified within the canonical PAZ domain using MEME Suite. Schematic representation (top) shows the approximate location of four conserved motifs (Logos 1–4) along the PAZ region. Sequence logos illustrate consensus motifs (below logos) and their relative frequency in proteins where the PAZ domain was detected (PAZ-found) versus those where it was not (PAZ-not-found). (**b**) Structural comparison of PAZ domains across Dcr categories. The full-length AlphaFold model of a canonical Dcr (SAM06846.1, Dicer-like 1 from *Absidia glauca*) is shown on the left, with the PAZ domain highlighted in yellow. Right panel: Representative AlphaFold structural models of PAZ domains from non-canonical categories A (KAF7757193.1, Dicer-like 1 from *Entomophthora muscae*), B (KAH8912380.1, unnamed protein from *Coniochaeta sp*. PMI_546), C (KAF8931068.1, Dicer-like 1 from *Dissophora ornata*), D (KAF1821536.1, unnamed protein from *Dissoconium aciculare*) and E (EGD96414.1, Dicer-like 1 from *Trichophyton tonsurans*), annotated with TM-score values and presence/absence of a recognizable cPAZ domain. PAZ regions in non-canonical Dcrs are color-coded according to their category (A–E). Structural alignment was performed using the PAZ domain of SAM06846.1 as reference to compare the spatial conservation of PAZ-like regions across non-canonical Dcrs. (**c**) Boxplot of TM-scores comparing structural similarity of PAZ domains across canonical and non-canonical Dcr categories. Canonical domains show consistently higher structural conservation. (**d**) Sensitivity comparison of the canonical PAZ HMM (PAZ) versus the fungal-specific FPAZ-HMM (FPAZ) across Eukaryotic groups.

We explored the possibility of a diverged fungal PAZ (fPAZ) in Dcr proteins lacking a detectable cPAZ. To address this idea, we compared the sequence and structural features of the cPAZ, present in canonical and non-canonical A and C Dcrs, with those of the putative fPAZ, which should be found in non-canonical B, D and E proteins. In proteins without a cPAZ, the fPAZ was defined as the region between the DUF283 and RNAse III domains. Using MEME [52], we identified four conserved motifs shared across both cPAZ and fPAZ domains. These motifs, designated Logo-1 through Logo-4, represent conserved sequence elements ordered from N- to C-terminal within the PAZ region (Fig. 5A). The logos revealed moderate to high positional conservation of aminoacidic residues across the motifs. Logo-1 displays highly conserved arginine (R) residues, and a conserved phenylalanine (F) at position 8. These residues reflect the characteristic composition of the 3’-end binding pockets in PAZ domains, where aromatic residues enable base stacking, and positive residues contribute to electrostatic interactions with the RNA [19], [53]. Logo-2, similar to Logo-1, contains conserved F and tyrosine (Y) residues, followed by a stretch of basic residues, further supporting a conserved RNA-binding role. Logo-3 features several polar and charged residues, including K, D, N and Q, which are likely involved in maintaining the OB-fold structure of the PAZ domain via hydrogen bonding and overall stabilization. The presence of conserved K and N residues in the central region may also contribute to interactions with the phosphate in the RNA. Logos 1–3 were strongly associated with the cPAZ, being identified in over 80 % of cPAZ-containing proteins, but in fewer than 20 % of the sequences containing only an fPAZ. In contrast, Logo-4 was more broadly conserved across fungal Dcrs. It was present in 99.67 % of the cPAZ sequences and in 56.71 % of the fPAZ sequences. This motif is enriched in hydrophobic amino acids (F, I, P, A, L, M), consistent with a helical or turn-forming structure. Given its composition, Logo-4 likely localizes to the connector helix that links the PAZ to the first RNAse III domain, a region well-known to be essential for maintaining the spatial configuration required for precise RNA cleavage by Dcr [54]. Mapping these four motifs onto the three-dimensional structure of a representative canonical PAZ domain confirmed their spatial clustering at the RNA-binding interface (Supplementary Fig. 3). In particular, the motifs localize around the region that interacts with the 3′ end of the dsRNA, supporting their proposed role in RNA anchoring and stabilization. The spatial proximity of Logo-1 and Logo-2 to the RNA entry groove aligns with their enrichment in basic and aromatic residues, while Logo-4 occupies a linker region adjacent to the RNase III domain, consistent with a role in domain coordination and structural integrity. This structural mapping provides further support for the functional relevance of these motifs in mediating RNA binding across diverse fungal Dcrs.

Next, we assessed the structural similarity of the cPAZ and divergent fPAZ domains. As a reference, we used the cPAZ domain from a representative canonical Dcr (Dcl1 from *Absidia glauca*). Structural comparisons revealed a high degree of similarity between this reference and the cPAZ domains of non-canonical Dcrs A (Dcl1 from *Entomophthora muscae,* TM-score of 0.821) and C (Dcl1 from *Dissophora ornata,* TM-score of 0.828). Although lower, structural similarity scores between the reference cPAZ and the putative fPAZ domains found in non-canonical Dcrs B, D and E (TM-scores of 0.678, 0.673 and 0.683, respectively), still indicate substantial structural conservation. This pattern of conserved fold architecture extends across the broader set of structural models analyzed (Fig. 5C).

Considering the high sequence divergence of fungal PAZ domains, and the under- representation of fungal Dcrs in the current PAZ HMM model, we generated a custom HMM-profile (FPAZ-HMM) based on a multiple sequence alignment of both cPAZ and structurally inferred fPAZ domains from our dataset (Supplementary File 11). We used this profile to search for PAZ domains in Dcr-annotated proteins in UniProtKB. Compared to the current PAZ HMM, the FPAZ-HMM profile demonstrates markedly improved sensitivity for detecting PAZ domains in fungal Dcrs, while maintaining comparable performance in Metazoa and Plantae (Fig. 5D). Specifically, the canonical PAZ model detected only 9 % of fungal PAZ domains, whereas FPAZ-HMM recovered 97.9 % of them, matching the sensitivity observed for Metazoan (98.8 %) and plant (96.5 %) Dcrs. These results demonstrate that incorporating divergent fungal sequences into the training alignment substantially improved sensitivity without compromising model performance in other eukaryotic groups.

Importantly, FPAZ-HMM was specifically trained to recognize PAZ domains associated with Dicer proteins-only, as other PAZ-containing proteins (PIWI, AGO) were intentionally excluded from the alignment used for model construction. Testing 236 proteins named AGO or ARGONAUTE found in the NCBI, we identified 0 discoveries of the FPAZ-HMM domain (false positives), suggesting high precision. In contrast, current general PAZ HMMs typically include PAZ domains from both Dcr and AGO proteins. These results support using of FPAZ-HMM as a robust and lineage-inclusive tool for detecting Dcr PAZ domains across eukaryotes and underscores the extensive sequence variability of PAZ domains across fungal clades.

### 2.7 Structural and functional evidence support conserved RNA-binding activity of fungal PAZ domains

**Fig. 6:**
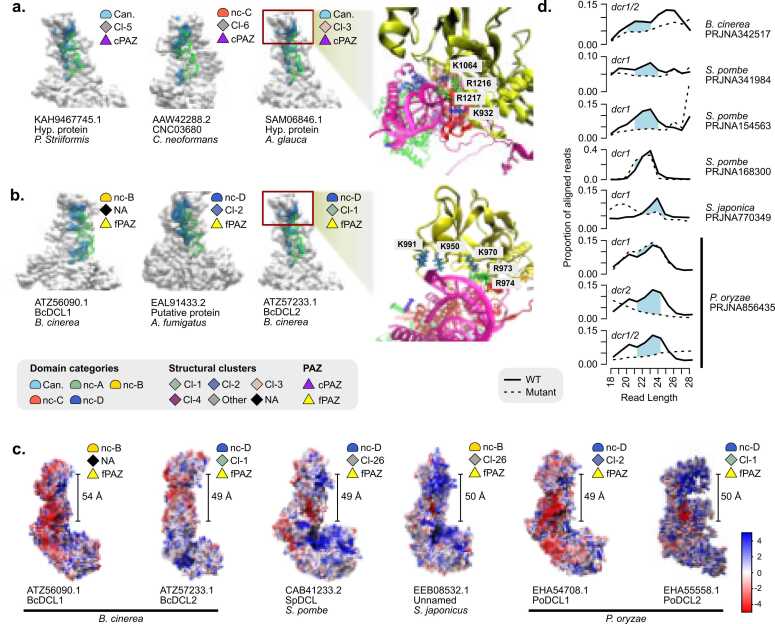
Structural and functional characterization of RNA-binding interfaces in canonical and non-canonical fungal Dcr proteins. (a–b) Surface representations of models of full-length Dcr proteins (gray) in complex with a pre-cleaved dsRNA substrate that was obtained from the PDB model of *D. melanogaster* Dicer (pdb_00007w0f) (green), showing the RNA-binding interfaces (blue). (**a**) Canonical Dcr proteins containing a canonical PAZ domain (cPAZ), grouped by structural cluster and domain category (Can.: canonical, nc-A-E: non-canonical A-E). (**b**) Non-canonical Dcrs possessing fungal-specific PAZ regions (fPAZ), grouped by structural cluster and domain category. Representative residues involved in interaction with RNA (lysines and arginines) are shown in atomic detail, with the side chains located within 3 Å of the RNA. Red boxes highlight the cPAZ or fPAZ region that anchors the 3′ end of the dsRNA. "Other" includes proteins belonging to minor structural clusters outside the four major groups. "NA" includes proteins that did not meet the clustering thresholds (≥90 % TM-score similarity and pLDDT ≥70). (c) Electrostatic surface potential is shown for each model, with red indicating negative charge and blue indicating positive charge (scale in kT/e). The measured distance between the fPAZ region and the RNase III catalytic site (in Å) is shown, suggesting its possible role in determining the length of small RNA (sRNA). (**d**) Read length distribution of the sRNA libraries. Solid lines represent WT, dashed lines correspond to dcr mutants. Light blue peaks indicate sRNA species lost in the mutant background.

To gain insights into the functionality of the canonical and divergent fungal PAZ domains, we analyzed structural models of full-length canonical and non-canonical Dcrs predicted in complex with a pre-cleaved dsRNA substrate that was obtained from the PDB model of *D. melanogaster* Dicer (pdb_00007w0f) (Fig. 6A, B). In these models, the dsRNA (green) interacts with the Dcr surface (grey), with key RNA-interacting residues highlighted in blue. In Dcrs with cPAZ, such as those from *Puccinia striiformis, Cryptococcus neoformans* and *Absidia glauca*, the PAZ domain and surrounding regions form a well-defined RNA-binding pocket. Basic residues, particularly K and R were consistently positioned within 3 Å of the dsRNA, forming a positively charged cleft that anchors the 3’ end of the RNA duplex (Fig. 6A, right). This configuration mirrors previously described PAZ-RNA interactions in plant and animal systems.

Remarkably, similar interaction interfaces were observed in non-canonical Dcrs lacking a canonical PAZ domain (cPAZ), but containing a structurally defined fungal-specific PAZ (fPAZ), including Dcl1 and Dcl2 from *Botrytis cinerea* and a Dcr from *Aspergillus fumigatus*. These proteins display conserved K and R residues positioned in close proximity to the RNA substrate, supporting the idea that key electrostatic and structural features necessary for RNA anchoring are retained, even in the absence of canonical sequence motifs. To assess the conformational stability of these interactions, we conducted molecular dynamics simulations on a representative set of Dcr–RNA complexes. Across all variants analyzed, including canonical, non-canonical C, and fPAZ-containing non-canonical B and D, RMSD trajectories stabilized within the first 10–20 nanoseconds, indicating that the RNA-binding interface, particularly the PAZ or PAZ-like domain, maintains structural integrity throughout the 50-nanosecond simulations (Supplementary Fig. 4). These results suggest that the fPAZ fold may act as a stable RNA-interacting module across divergent fungal Dcrs.

To further explore the functional potential of the fungal PAZ regions, we analyzed the electrostatic surface distribution of representative Dcr proteins for which a function in sRNA biogenesis has been evaluated using mutants and sRNA sequencing. These include Dcl1 and Dcl2 from *B. cinerea*, Dcr1 from *S. pombe*, Dcr1 from *S. japonicus*, and Dcl1 and Dcl2 from *P. oryzae* (Fig. 6C). In these non-canonical Dcrs, the dsRNA-binding pocket displayed a predominance of positively charged residues, consistent with a role in RNA anchoring (Fig. 6C). Additionally, we measured the distance between the fPAZ domain and the RNAse III catalytic center, finding it to be approximately 50–55 Å, comparable to that reported in human Dicer [10]. These structural configurations support the model in which PAZ domains act as a molecular ruler, securing the 3’end of the RNA and enabling precise cleavage by the RNAse III domains.

Finally, we compared sRNA length profiles from wild-type and mutant strains with disrupted dcr genes (Fig. 6D). In all cases examined, dcr mutation resulted in clear losses of distinct sRNA size classes, as indicated by the disappearance of specific peaks. These results support the idea that non-canonical Dcrs may contribute to sRNA biogenesis and strongly suggest that the fPAZ domain could play a critical role in RNA processing, despite its sequence divergence from canonical PAZ domains.

## 3 Discussion

Fungi represent a major eukaryotic lineage positioned between plants and animals and exhibiting exceptional molecular and ecological diversity. As RNAi originated early in eukaryotic evolution, understanding the structural diversity of Dcr proteins in fungi is essential for understanding how RNAi pathways have been maintained, adapted, and diversified across eukaryotes. To our knowledge, the dataset presented in this study represents the most extensive survey of fungal proteomes derived from annotated and reference-quality genomes to date, comprising 1592 proteomes representing 1422 species across nine phyla and 44 taxonomic classes. While thousands of fungal genomes are publicly available, many are incomplete or lack standardized annotations, which limits their utility for comparative analyses of protein-coding genes such as Dcrs. Notably, our sampling includes a broader representation of non-Dikarya fungi compared to previous studies [21], [34], particularly within Mucoromycota and Microsporidia. This improvement reflects the increasing contribution of large-scale sequencing efforts such as the Joint Genome Institute’s 1000 Fungal Genomes Project (https://mycocosm.jgi.doe.gov/mycocosm/home/1000-fungal-genomes). Despite this broader coverage, several phyla are still poorly represented in our dataset, including Zoopagomycota (20 proteomes), Chytridiomycota (19 proteomes), Blastocladiomycota (2 proteomes), and Olpidiomycota (1 proteome), while other phyla are entirely absent. These limitations highlight the continued need to incorporate non-Dikarya and ecologically diverse fungal lineages into genome sequencing initiatives, to enable a more complete understanding of fungal molecular diversity.

Our survey of fungal Dcrs identified proteins broadly across fungal clades in the dataset, confirming that this machinery is widely retained across fungi, as others have found [21]. Nevertheless, we found a relevant part of fungal species (∼10 %) with no Dcr identified in their proteomes, according to our pipeline. While the loss of Dcrs is well established in budding yeasts within the Saccharomycotina and some Basidiomycota lineages [22], [28], broader surveys have shown that such losses may also occur sporadically across the fungal kingdom [37], [33]. However, the extent of Dcr loss in other fungal groups remains less clear, largely due to the underrepresentation of non-Dikarya fungi in genomic datasets. In our analysis, most proteomes lacking detectable Dcrs belong to Saccharomycotina, consistent with previous reports. However, we also identified apparent Dcr losses in non-Dikarya phyla. While these absences require further validation, they raise the possibility that some fungal lineages may have evolved alternative RNAi pathways operating independently of Dcr proteins, or that the RNAi pathway itself may have been lost in these groups. This contrasts with animal and plant systems, were the loss of Dcr typically results in severe developmental defects or lethality [55], [56] and suggests that fungi might possess a more flexible RNA silencing machinery. Consistent with this idea, evidence of Dcr-independent sRNA-processing by other members of the RN*Ase*III family has been reported in fungi, as is the case of MRPL3 in *Neurospora crassa,* or R3B2 in *Mucor lusitanicus* [51], [57]*.* Fungal genomes encode a diverse array of RNases III which could play conceivable roles in RNAi. For example, our in-depth analysis of RNAse III-containing proteins uncovered a small number of potential DROSHA-like candidates. However, the general absence of the characteristic domain architecture typically associated with DROSHA suggests that these proteins may be highly divergent and thus require more targeted, in-depth analyses for definitive identification.

We found four major structural clusters of fungal Dcr proteins, although their specific functional properties remain unexplored. Within Dikarya, members of distinct Dcr clusters are found, and several species possess two or three paralogs that map to different clusters, suggesting lineage-specific expansions. Such diversification may be related with the expansion and specialization of RNAi functions in these organisms, potentially reflecting evolutionary pressures to fine-tune RNAi responses to distinct developmental or ecological contexts. Notably, sequence-diverging PAZ domains appear to be widespread among the species analyzed, whereas cPAZ domains are restricted to a limited number of species, mostly within Mucoromycota and Basidiomycota. Considering that ACE analysis supports that cPAZ was likely present in the last common ancestor of fungal Dcrs, this limited representation implies lineage-specific retention, which could be attributed to selective pressures to maintain a more precise or processive sRNA cleavage in these lineages. Both Mucoromycota and Basidiomycota comprise species engaged in complex ecological interactions, including mutualistic symbioses, such as Mycorrhizal associations, and pathogenic lifestyles. In these contexts, sRNA-mediated communication between fungi and their plant hosts may play a pivotal role in modulating host responses and facilitating successful colonization.

Our phylogenetic analysis revealed that Dcr naming in databases has not been consistently applied across fungal lineages. Proteins carrying common names (Dcr1/Dcl1 or Dcr2/Dcl2) often appear in different clades, indicating that these labels do not accurately reflect true evolutionary or functional relationships. For example, proteins annotated as Dcr1/Dcl1 are distributed across Ba2 and Ba3 in Basidiomycota, which form separate phylogenetic and structural clusters. Similar patterns are observed for other Dcr annotations. These observations highlight that current Dcr naming conventions do not represent the structural and phylogenetic diversity of fungal Dcr proteins, underscoring the need for a more systematic, structure- and phylogeny-informed classification framework. Considering that Dcr naming in organisms with multiple Dcrs such as plants or insects is related to their biochemical functions, and that protein function is primarily dictated by structure, we propose a Dcr annotation scheme based on the four structural clusters identified in our analysis, which capture the core architectural diversity of fungal Dcrs. This scheme assigns each validated Dcr a short identifier constructed with the prefix Dcf (Dicer fungi), and the structural cluster number (e.g., Dcf2 for Dcr from structural cluster 2). We have provided suggested names based on this annotation for all validated Dcrs in this work on Supplementary Table 2. We envision that this format could serve as a foundation for future integration of fungal Dcr annotations in public repositories.

In fungi, the PAZ domain has traditionally been considered dispensable for Dcr function, since various well-characterized fungal models, including *Schizosaccharomyces pombe, Neurospora crassa*, *Trichoderma atroviride*, *Aspergillus fumigatus*, or *Botrytis cinerea,* harbor non-canonical Dcrs that lack detectable PAZ domains but remain functional in sRNA production [22], [32], [47], [48], [49], [50], [51]. However, our structural analyses challenge this notion. Many of these non-canonical Dcrs retain a PAZ-like fold capable of anchoring dsRNA despite low sequence similarity to canonical PAZ domains. This finding suggests that structural conservation, rather than primary sequence similarity, may underline the preservation of PAZ functionality in fungi. Although these computational analyses provide compelling structural evidence supporting a potential RNA binding function for fPAZ, we acknowledge that these findings remain predictive and require experimental validation. Future studies could address this by performing RNA binding assays using purified fPAZ domains or in vitro dicing assays with recombinant fungal Dcrs containing fPAZ.

Detailed structural comparisons revealed strong conservation of R and K residues in the binding pocket pointing to a conservation of the dsRNA-protein interface. However, we found that the highly hydrophobic Logo-4 motif is much more conserved in fungal Dcrs. This hints that the overall structure of the PAZ domain is more conserved among fungi, while the actual binding interface could be more lineage specific (Logos-1, −2, −3). Consistent with previous studies [58], we found that PAZ-like domains in fungal Dcrs conserve the R and K residues critical for 3′ end binding. Amino acids like E, R, and K are often involved in ionic interactions and hydrogen bonding, essential for dsRNA binding. Y and F contribute to hydrophobic interactions, stabilizing the RNA-protein complex. The identification of these conserved residues and their role in ionic interactions, hydrogen bonding, and hydrophobic stabilization strengthens the hypothesis that even non-canonical PAZ domains can perform essential functions. However, experimental approaches are necessary to confirm whether these predicted interactions translate into functional conservation. For instance, residues such as R and K, which mediate 3′ end binding, as well as other amino acids involved in electrostatic and hydrophobic interactions, are promising candidates for site-directed mutagenesis to assess their specific contribution to RNA binding and cleavage efficiency. These experiments would help clarify whether structurally conserved PAZ-like folds can fully compensate for the absence of canonical PAZ domains in non-canonical Dcrs. Moreover, identifying lineage-specific conservation patterns, such as the highly preserved hydrophobic Logo-4 motif, could inform the design of small molecule inhibitors or RNA mimetics that selectively disrupt dsRNA recognition in certain fungal groups. This may have practical implications for biotechnological or agricultural applications, such as developing of fungal RNAi-based tools for gene silencing or plant-pathogen interaction control. Ultimately, resolving how these motifs contribute to RNA processing in structurally divergent Dcrs will enhance our understanding of fungal RNAi flexibility and evolutionary innovation.

Our analyses suggest that fungal Dcr proteins may retain essential RNA-binding capabilities through structurally conserved domains, even when canonical sequence motifs are not detectable. This observation raises the possibility that domain absence inferred from sequence similarity does not necessarily imply functional loss. Instead, our in silico results indicate that structural and electrostatic properties potentially supporting RNA-binding could persist despite extensive sequence divergence, hinting at the evolutionary resilience of these interfaces.

The difficulty of sequence-based domain models (e.g., InterProScan, Pfam HMMs) to detect PAZ-like regions in fungal Dcrs exemplifies this limitation. These tools rely on hidden Markov models trained primarily on canonical sequences from animals and plants and may fail to detect highly divergent variants that preserve structural and functional features despite lacking recognizable sequence similarity. This issue has also been reported in recent work using structure-based HMMs [59]. As a result, structurally conserved domains can remain undetected, leading to underestimation of functional conservation across divergent eukaryotic lineages [39].

Although our primary goal was to improve domain detection in fungal Dcrs, FPAZ-HMM also demonstrated robust performance in Dicer proteins from plants and metazoans available in NCBI. Comparative evaluation against the canonical PAZ model (PF02170) showed high sensitivity for known Dcrs, while avoiding annotation of PAZ in AGO proteins. This precision and sensitivity across diverse fungi suggests this may be valuable to search for Dcr in other less-studied eukaryotic lineages, such as protists, microalgae, or eukaryotic parasites. Such taxa often exhibit high sequence divergence and are underrepresented in canonical domain models, a challenge previously described in other divergent lineages using structure-informed HMMs [60], [61]. Our strategy demonstrates that structurally informed HMM profiles which include more diverse proteins can improve annotation quality in such groups, uncovering hidden domain conservation and guiding functional predictions in underexplored branches of the eukaryotic tree. Overall, this highlights FPAZ-HMM as a useful complementary profile to canonical PAZ.

Beyond their methodological relevance, fungal Dcrs may represent an informative model for exploring modular protein evolution, in which domains can be lost, diverge, or become structurally reconfigured while potentially retaining aspects of their function. While our analyses provide in silico evidence consistent with such modular flexibility, experimental validation will be essential to confirm these hypotheses. This apparent plasticity could reflect selective pressures to fine-tune or adapt RNAi responses to diverse ecological contexts, pressures that may have also shaped other multidomain regulatory proteins across eukaryotes. By combining phylogenetic, structural, and motif-based analyses, this study illustrates how computational approaches can help uncover potential functional conservation within rapidly evolving systems.

In addition to its evolutionary implications, the identification of structurally conserved RNA-binding domains in non-canonical fungal Dcrs may also hold practical significance. Several fungal species harboring such divergent Dcrs, including *Botrytis cinerea*, *Trichoderma atroviride*, and *Puccinia striiformis*, are agriculturally relevant pathogens or symbionts, raising possibilities about how Dcr diversification might influence interspecies communication. From a biotechnological perspective, understanding how fungal RNAi machinery tolerate or compensate for domain loss could guide the development of fungal-specific gene silencing tools, such as minimal RNAi constructs for functional genomics or novel strategies for crop protection through interspecies RNAi.

## 4 Materials and methods

### 4.1 Recovery and quality assessment of fungal proteomes

We retrieved a total of 1592 annotated and reference fungal proteomes from the NCBI (data available up to November 2022), corresponding to 1418 unique species and 170 duplicated species from different strains. We used the Entrez esearch tool via its command line interface [62], with the query "Fungi"[Organism] OR fungi[All Fields]) AND (fungi[filter] AND "representative genome"[filter] AND (all[filter] NOT anomalous[filter] AND all[filter] NOT partial[filter]) AND "has annotation"[Properties]', applying filters to select only representative genomes with available annotations, while excluding anomalous or partial assemblies.

We used BUSCO (Benchmarking Universal Single-Copy Ortholog assessment) [42] v.5.3.2 to evaluate the completeness of proteomes. We first applied the universal_odb10 and fungi_odb10 dataset, followed by additional phylum-specific datasets when available, including asomcyota_odb10, basidiomycota_odb10, microsporidia_odb10, and mucoromycota_odb10. All retrieved proteomes were retained for downstream analyses, regardless of whether their BUSCO completeness was above or below 90 %. We made this decision to maximize the detection of Dcr proteins, ensuring that potentially relevant sequences from less complete proteomes were not excluded. For example, some fungal groups, such as Cryptomycota and Olpidiomycota, are known to include obligate parasites, which may require a specialized dataset to accurately assess completeness.

We labeled each proteome with its corresponding BUSCO completeness score, allowing its quality to be tracked throughout the subsequent analyses. This annotation ensured that for every Dcr protein identified in later steps, the completeness of its source proteome was known. We retained this information to assess whether proteome completeness had any impact on Dcr detection and to provide a transparent evaluation of the dataset composition.

### 4.2 Recovery of profile Hidden Markov Model and identification of putative Dcr proteins

We retrieved a set of profile Hidden Markov Models (HMMs) representing highly conserved Dcr domains within eukaryotic species from the Interpro database (ebi.ac.uk/interpro). These HMMs have been used in previous Dcr studies [22], [43], [63] and include PF00270/IPR011545 (representing the DEAD/DEAH box helicase subdomain), PF04851/IPR006935 (representing ResIII helicase subdomain), and PF00271/IPR001650 (representing Helicase C terminal helicase subdomain), PF00636/IPR000999 (representing RNase III domain), PF02170/IPR003100 (representing PAZ domain), PF03368/IPR005034 (representing DUF283/Dicer dimerization domain), and PF00035/IPR014720 (representing Double-stranded RNA binding motif domain). We scanned the fungal proteomes with the HMMER v.3.3.1 toolkit [64] to identify putative Dcr proteins. The thresholds for detection of a domain were set at an E-value ≤ 10^−3^, and only proteins containing at least one RNase III domain (PF00636/IPR000999) were retained for further analysis.

### 4.3 InterProScan analysis

Protein sequences were analyzed using InterProScan v.5.44 [65], executed from the command line with default parameters.

### 4.4 Isoform identification and removal of annotation redundancies

To avoid overestimating the number of Dicer proteins per species due to redundant annotations or isoform variants, we implemented a filtering strategy based on the integration of genomic, proteomic, and external database information. For each identified protein, we retrieved the following metadata: NCBI protein accession, sequence length, annotated locus, GeneID, and associated UniProt accession (when available).

Sequences sharing the same GeneID and locus but differing in their protein identifiers were considered potential isoforms and were retained as separate entries if they exhibited differences in their primary amino acid sequence (e.g., variation at the N- or C-termini). In contrast, when two or more entries belonged to the same species, showed 100 % identity in their amino acid sequences, and shared the same UniProt ID, they were classified as annotation duplicates. In these cases, we retained a single representative copy.

### 4.5 In silico protein structure prediction

To predict the three-dimensional structures of the Dcr proteins, we used the local version of Colabfold [66] v1.5.5, a Google Colab implementation of Alphafold2 [41]. In the first step, multiple sequence alignments (MSA) were generated against all available databases using the “DB load mode 3” setting, which preloads all sequence databases into memory to speed up alignment searches. We executed this process using the following command: colabfold_search --db-load-mode 3 --threads 54 dcr_sequences.fasta database a3m_output. Subsequently, we predicted the protein structures using the same software with the command: colabfold_batch --num-models 1 --num-relax 0 msas_input/ pdb_output/. To reduce computational time, we only generated one structural model per protein, and no structural relaxation was performed. We selected protein models with a predicted Local Distance Difference Test (pLDDT) score ≥ 70 for further analysis, ensuring the inclusion of structures with high predicted accuracy for downstream applications. This single-model strategy allowed us to maintain consistency across > 2900 proteins and to focus on high-confidence predictions suitable for comparative structural and electrostatic analyses. Although additional relaxation or multiple-seed modeling can sometimes improve borderline cases, the top-ranked AlphaFold models already provided sufficient accuracy for the purposes of this study.

### 4.6 Electrostatic surface calculations

To investigate the electrostatic surface distribution of selected Dicer proteins, we applied a two-step computational workflow consisting of structure preparation and electrostatic potential calculation. Protein structures were preprocessed using PDB2PQR [67], which assigns atomic charges and radii according to the PARameters for Solvation Energy (PARSE) force field and determines protonation states at pH 7.0 using PROPKA [68], [69]. This preprocessing ensures consistent treatment of ionizable residues, addition of hydrogens, and optimization of side-chain orientations for electrostatic calculations. Electrostatic potentials were then computed using the Adaptive Poisson–Boltzmann Solver (APBS) [70]. We performed the calculations using the linearized Poisson–Boltzmann equation (LPBE), with the solute and solvent dielectric constants set to 2.0 and 78.54, respectively. We used a solvent probe radius of 1.4 Å, temperature of 298.15 K, and ion-accessibility parameters (swin = 0.3 Å, sdens = 10.0). We set surface and charge discretization methods to smol and spl2, respectively. We set the boundary condition to sdh (single Debye–Hückel), and we automatically generated the electrostatic grid to encompass each protein’s structure with sufficient resolution. Electrostatic potential maps were output in OpenDX format and used to visualize surface charge distributions, with a focus on the double-stranded RNA binding regions. Total electrostatic energies were also computed but not directly analyzed.

### 4.7 Generation of protein similarity networks and protein structural networks

We generated a sequence similarity network using the Sequence Similarity Network pipeline v.1.0.0 (https://github.com/MiguelMSandin/SSNetworks). In brief, this pipeline performs a local pairwise alignment of sequences using BLASTn to calculate similarities between all sequences in the dataset. We processed the resulting pairwise similarities to remove reciprocal hits and self-hits, ensuring a clean input for network construction. The identity threshold was based on preliminary testing, observing more stringent identity cutoffs (> 60 %) resulted in extensive network fragmentation, and lower thresholds (< 40 %) clumped protein into to a unique connected component. We chose an intermediate value of 50 % for identity threshold, reflecting these concerns. We used a strict coverage threshold of 80 %, ensuring we retained only alignments spanning most of the protein length and minimizing artifactual associations based on short, conserved motifs. For the protein structural network, we aligned the obtained protein structures in PDB format using mTM-align [71]. We generated the structural network considering TM-scores between nodes of 90 % or more. We used Cytoscape [72] v.3.10.0 to visualize and analyze the networks.

### 4.8 Phylogenetic tree generation

Proteins from each of the 149 clusters identified through the sequence similarity network analysis (described in the previous paragraph) were independently aligned using T-Coffee [73] v13.45.0 with default parameters, as this method provides improved accuracy for aligning divergent sequences within each cluster. The resulting 149 individual alignments were then merged into a single global alignment through profile alignment using ClustalW [74] v.2.1 with the -profile option, which efficiently integrates pre-aligned sequence blocks. The global protein alignments were trimmed using ClipKit [75] v2.1.1 with default parameters. We inferred the best-fit model and maximum-likelihood phylogenetic trees from the trimmed sequence alignments using IQ-TREE [76] v2.2.5 with the following parameters: -B 1000 -m MFP -nt AUTO.

### 4.9 Alignment quality assessment

To evaluate the reliability of multiple sequence alignments across major Dcr clades from Fig. 2, we used AliStat v1.15 [77]. AliStat was executed in amino acid mode (AA; data type = 6) using the -b option to compute the completeness score for the alignment (C_a_), number of unambigous characters in a row of the alignment (C_r_), completeness score for individual sites (C_c_) and completeness score for pairs of sequences (C_ij_) for each alignment (e.g.,./alistat alignment.fasta 6 -b). Summary statistics were compiled across clades into a single dataset to quantify alignment quality and completeness.

### 4.10 Ancestral reconstruction

We used the fitmk function of the phytools [78] package to fit a discrete Markov model for the ancestral reconstruction of the Dcr proteins, taking as input the phylogenetic tree generated above. In this case, we used the "Equal Rates" (ER) model, which assumes equal transition rates between states, with the following command: fitER = fitMk(midroot_tree, PAZ_level, model = "ER"). The anc.recon function was used based on the fitted model, and we performed ancestral reconstruction using the marginal method to infer the most probable states at the ancestral nodes of the tree, using the command: fit.marginal = ancr(fitER, type = "marginal").

### 4.11 Horizontal gene transfer analysis

To assess the potential occurrence of horizontal gene transfer (HGT) among fungal Dcrs, we analyzed Dcr sequences using HGTector [79] v2.0b3. The search module of HGTector was executed with DIAMOND [80] against a pre-built NR reference database retrieved in January 2023 from https://github.com/qiyunlab/HGTector/blob/master/doc/database.md#pre-built-databases, and including 40,310 genomes. We run the ‘analyze’ command using default parameters, except for the --self parameter, which was set to 4751 (NCBI taxonomy ID for Fungi) to filter intra-fungal homologs.

### 4.12 Docking and molecular simulations

We performed docking of the Dcr-RNA complex using HDOCK [53] v1.0. Protein models of Dcr, obtained from the ColabFold analysis, were provided in PDB format, and were used along with the dsRNA derived from the Drosophila Dicer2 PDB model (7W0F). We performed *ab initio* docking, defining the residues in the receptor (Dcr) and ligand (RNA) where preferential binding was expected. For the molecular dynamics simulations, we used the CHARMM36 [81] force field to construct the protein structure file (PSF) for both protein and dsRNA. The simulation workflow consisted of three stages. First, energy minimization was performed using 20,000 steps to remove steric clashes and stabilize the initial system using a restriction of 1 kcal/mol. Next, an NVT equilibration phase was conducted for 0.25 ns constraints applied to the protein and dsRNA to maintain structural integrity during thermal adjustments (1 kcal/mol). Finally, production simulations were carried out in two phases: 50 ns simulations with dsRNA restrictions maintained, followed by an additional 50 ns simulations with all constraints released to allow full system flexibility. Each molecular simulation was performed in three independent replicates to assess the reproducibility and stability of the results. We generated all molecular dynamics steps using NAMD3 [82]. The equations of motion were integrated with a 2-fs time step using the Verlet algorithm. Langevin dynamics (damping coefficient of 1 ps) and the Nosé–Hoover Langevin piston method was employed to maintain constant temperature (310 K) and pressure (1 atm). Long-range electrostatic interactions were calculated using the Particle Mesh Ewald (PME) method, and van der Waals forces were computed with a cutoff of 12 Å.

### 4.13 Motif discovery in protein sequences

We performed motif discovery using the Multiple Em for Motif Elicitation (MEME) tool v. 5.5.7 from the MEME suite [52]. We ran MEME locally with the options -protein -mod zoops -nmotifs 4 -minw 6 -maxw 50. We used the obtained motifs to scan the sequences with the Find Individual Motif Occurrences (FIMO) from the MEME suite. We ran FIMO with default parameters.

### 4.14 Construction of a custom HMM profile for the PAZ domain

To generate a high-confidence HMM profile of the PAZ domain, we selected 2452 PAZ-containing regions from fungal Dicer proteins, including both canonical (cPAZ) and non-canonical (fPAZ) representatives identified in our dataset. We first aligned sequences separately using MAFFT [83] v7.505 with the --auto option, visually inspecting alignment quality to confirm the presence of informative blocks. Alignment regions were trimmed using ClipKit v.1.3.0 in kpi (keep phylogenetically informative) mode, which retains sites contributing the highest phylogenetic signal while removing regions that have low residue conservation across homologous sequences. We then merged the resulting canonical and non-canonical alignments via profile-profile alignment using ClustalW v2.1. This combined alignment was once again trimmed with ClipKit to remove potential residual noise. We used the resulting filtered alignment to build a PAZ domain-specific HMM profile using hmmbuild from the HMMER suite v3.3. The final model consisted of 395 match states (effective sequence number = 24.25; relative entropy per position = 0.59), indicating moderate diversity and information content. Attempts to improve the entropy score using the --symfrac 0.7 option did not yield significant differences.

We evaluated the sensitivity (also called recall) of the model using hmmsearch against several curated datasets, including fungal Dicer proteins identified in our pipeline and representative Dicer-like proteins from plants and animals. True positives (TP) were defined as sequences successfully detected by the FPAZ-HMM model, and false negatives (FN) as Dicer sequences in the dataset that were not detected. Sensitivity was calculated as TP / (TP + FN), following the conventional formula. Although we did not formally assess precision, we verified that the model did not detect PAZ domains in a set of unrelated negative controls, including 236 ARGONAUTE proteins found in the NCBI and the ETHYLENE INSENSITIVE3-LIKE 1 (EIL1) transcription factor from *Arabidopsis thaliana*, supporting its discriminatory capacity.

### 4.15 Processing of sRNA-seq libraries

We performed a comprehensive search of publicly available sRNA-seq datasets in NCBI, utilizing the NCBI-SRA tools, eDirect, and custom python scripts using fungal-related terms (e.g., fungi, fungus, specific fungal genera) combined with sRNA-related keywords (e.g., miRNA, microRNA, milRNA, small RNA*, sRNA, siRNA, smRNA, epigen*, RNAi). We cross-referenced the publications matching the search criteria with NCBI sample databases (SRA, Bioprojects, Biosamples, and GEO datasets) to identify relevant datasets. We manually reviewed the entries to identify BioProjects containing libraries likely including fungal sRNA-seq data from wild-type or Dicer mutants and we downloaded the libraries using SRAtools. We processed the selected libraries using the YASMA small RNA analysis suite [84] (github.com/nateyjay/yasma). 3’ adapters were identified using YASMA-adapter and trimmed with cutadapt [85] with the following settings: cutadapt -a [adapter] --minimum-length 15 --maximum-length 50 -O 4 --max-n 0 --trimmed-only -o [out_file] [file]. We aligned trimmed sequences to the closest matching reference genome using YASMA-align, which mimics the unique-weighting approach found in ShortStack3 [86]. sRNAs were classified into general sizes using YASMA-size-profile.

### 4.16 Determination of molecular distances in protein structures

We used the Bio3D R library [87] to calculate the molecular distance between the N-terminus of the PAZ domain and the N-terminus of the RNAse IIIa domain. The protein structure, provided in PDB format, was imported to R using the read.pdb function and the coordinates of the alpha-carbon atoms corresponding to the N-terminal residues of the PAZ and RNAse IIIa domains were extracted. We calculated the Euclidean distance between these coordinates in Angstroms using the xyz.dist function in Bio3D.

## Author contributions

Designed research: L.M., N.R.J., E.A.V.; Performed research: L.M., J.C., P.V., B.V-V., F.G-T., I.O.; Analyzed data: L.M., J.C., P.V., B.V-V., F.G-T., I.O., J.A.R-P., J.P.C., C.M., V.C-F., N.R.J., E.A.V.; Wrote the paper: L.M., N.R.J., E.A.V.

## CRediT authorship contribution statement

**Cardenas Juan Pablo:** Writing – review & editing, Investigation, Formal analysis. **Ivana Orellana:** Writing – review & editing, Investigation, Formal analysis. **Rivas-Pardo Jaime Andres:** Writing – review & editing, Investigation, Formal analysis. **Boris Vidal-Veuthey:** Writing – review & editing, Investigation, Formal analysis. **Gonzalez-Toro Fabian:** Investigation, Formal analysis. **Jonathan Canan:** Writing – review & editing, Methodology, Investigation, Formal analysis. **Pablo Villalobos:** Writing – review & editing, Methodology, Investigation, Formal analysis. **Johnson Nathan:** Writing – review & editing, Writing – original draft, Methodology, Investigation, Formal analysis, Data curation, Conceptualization. **Elena A. Vidal:** Writing – review & editing, Writing – original draft, Supervision, Conceptualization. **Lorena Melet:** Writing – review & editing, Writing – original draft, Methodology, Investigation, Formal analysis, Data curation, Conceptualization. **Carol Moraga:** Writing – review & editing, Investigation, Formal analysis. **Castro-Fernandez Victor:** Writing – review & editing, Investigation, Formal analysis.

## Declaration of Competing Interest

The authors declare that they have no known competing financial interests or personal relationships that could have appeared to influence the work reported in this paper.

## Data Availability

Structural models of fungal Dicer-like (Dcr) proteins predicted by AlphaFold2, along with clade alignments and the custom Hidden Markov Model (FPAZ.HMM) used for PAZ domain identification, have been deposited in Figshare and are publicly accessible at: doi.org/10.6084/m9.figshare.28592981.

## References

[1] Lim L.P. (2005). Microarray analysis shows that some microRNAs downregulate large numbers of target mRNAs. Nature.

[2] Wilkins C. (2005). RNA interference is an antiviral defence mechanism in Caenorhabditis elegans. Nature.

[3] Volpe T.A. (2002). Regulation of heterochromatic silencing and histone H3 Lysine-9 methylation by RNAi. Science.

[4] Buck A.H. (2014). Exosomes secreted by nematode parasites transfer small RNAs to mammalian cells and modulate innate immunity. Nat Commun.

[5] Cai Q. (2018). Plants send small RNAs in extracellular vesicles to fungal pathogen to silence virulence genes. Science.

[6] Wang M. (2016). Bidirectional cross-kingdom RNAi and fungal uptake of external RNAs confer plant protection. Nat Plants.

[7] Martinez J., Patkaniowska A., Urlaub H., Lührmann R., Tuschl T. (2002). Single-stranded antisense siRNAs guide target RNA cleavage in RNAi. Cell.

[8] Schwarz D.S. (2003). Asymmetry in the assembly of the RNAi enzyme complex. Cell.

[9] Lau P.-W., Potter C.S., Carragher B., MacRae I.J. (2009). Structure of the human Dicer-TRBP complex by electron microscopy. Structure.

[10] Lau P.-W. (2012). The molecular architecture of human Dicer. Nat Struct Mol Biol.

[11] Wang (2009). Structural insights into RNA processing by the human RISC-loading complex. Nat Struct Mol Biol.

[12] Sinha N.K., Trettin K.D., Aruscavage P.J., Bass B.L. (2015). Drosophila Dicer-2 Cleavage Is Mediated by Helicase- and dsRNA Termini-Dependent States that Are Modulated by Loquacious-PD. Mol Cell.

[13] Sinha N.K., Iwasa J., Shen P.S., Bass B.L. (2018). Dicer uses distinct modules for recognizing dsRNA termini. Science.

[14] Welker N.C. (2011). Dicer’s helicase domain discriminates dsRNA Termini to promote an altered reaction mode. Mol Cell.

[15] Hiraguri A. (2005). Specific interactions between Dicer-like proteins and HYL1/DRB- family dsRNA-binding proteins in Arabidopsis thaliana. Plant Mol Biol.

[16] Qin H. (2010). Structure of the *Arabidopsis thaliana* DCL4 DUF283 domain reveals a noncanonical double-stranded RNA-binding fold for protein–protein interaction. RNA.

[17] Ota H. (2013). ADAR1 Forms a Complex with Dicer to Promote MicroRNA Processing and RNA-Induced Gene Silencing. Cell.

[18] Zhang H. (2002). Human Dicer preferentially cleaves dsRNAs at their termini without a requirement for ATP. EMBO J.

[19] Ma J.-B., Ye K., Patel D.J. (2004). Structural basis for overhang-specific small interfering RNA recognition by the PAZ domain. Nature.

[20] MacRae I.J. (2006). Structural Basis for Double-Stranded RNA Processing by Dicer. Science.

[21] Choi J. (2014). funRNA: a fungi-centered genomics platform for genes encoding key components of RNAi. BMC Genom.

[22] Drinnenberg I.A. (2009). RNAi in Budding Yeast. Science.

[23] Hu Y., Stenlid J., Elfstrand M., Olson Å. (2013). Evolution of RNA interference proteins dicer and argonaute in Basidiomycota. Mycologia.

[24] Li Y. (2021). A genome-scale phylogeny of the kingdom Fungi. Curr Biol.

[25] James T.Y., Stajich J.E., Hittinger C.T., Rokas A. (2020). Toward a Fully Resolved Fungal Tree of Life. Annu Rev Microbiol.

[26] Tedersoo L. (2018). High-level classification of the Fungi and a tool for evolutionary ecological analyses. Fungal Divers.

[27] Wijayawardene N.N. (2024). Classes and phyla of the kingdom Fungi. Fungal Divers.

[28] Dang Y., Yang Q., Xue Z., Liu Y. (2011). RNA Interference in Fungi: Pathways, Functions, and Applications. Eukaryot Cell.

[29] Nicolás F.E., Ruiz-Vázquez R.M. (2013). Functional diversity of RNAi-associated sRNAs in fungi. Int J Mol Sci.

[30] Nakayashiki H. (2005). RNA silencing in fungi: mechanisms and applications. FEBS Lett.

[31] Nicolás F.E., Moxon S., de Haro J.P., Calo S., Grigoriev I.V., Torres-Martínez S., Dalmay T. (2010). Endogenous short RNAs generated by Dicer 2 and RNA-dependent RNA polymerase 1 regulate mRNAs in the basal fungus Mucor circinelloides. Nucleic Acids Res.

[32] Weiberg A. (2013). Fungal Small RNAs Suppress Plant Immunity by Hijacking Host RNA Interference Pathways. Science.

[33] Billmyre R.B., Calo S., Feretzaki M., Wang X., Heitman J. (2013). RNAi function, diversity, and loss in the fungal kingdom. Chromosome Res.

[34] Huang Q. (2018). Evolution of Dicer and Argonaute orthologs in microsporidian parasites. Infect Genet Evol.

[35] Catalanotto C. (2004). Redundancy of the Two Dicer Genes in Transgene-Induced Posttranscriptional Gene Silencing in *Neurospora crassa*. Mol Cell Biol.

[36] Kadotani N., Nakayashiki H., Tosa Y., Mayama S. (2004). One of the Two Dicer-like Proteins in the Filamentous Fungi Magnaporthe oryzae Genome Is Responsible for Hairpin RNA-triggered RNA Silencing and Related Small Interfering RNA Accumulation. J Biol Chem.

[37] Chang S.-S., Zhang Z., Liu Y. (2012). RNA Interference Pathways in Fungi: Mechanisms and Functions. Annu Rev Microbiol.

[38] De Haro J.P. (2009). A Single *dicer* Gene Is Required for Efficient Gene Silencing Associated with Two Classes of Small Antisense RNAs in *Mucor circinelloides*. Eukaryot Cell.

[39] Kress A., Poch O., Lecompte O., Thompson J.D. (2023). Real or fake? Measuring the impact of protein annotation errors on estimates of domain gain and loss events. Front Bioinform.

[40] Zapletal D., Kubicek K., Svoboda P., Stefl R. (2023). Dicer structure and function: conserved and evolving features. EMBO Rep.

[41] Jumper J. (2021). Highly accurate protein structure prediction with AlphaFold. Nature.

[42] Manni M., Berkeley M.R., Seppey M., Simão F.A., Zdobnov E.M.B.U.S.C.O. (2021). update: novel and streamlined workflows along with broader and deeper phylogenetic coverage for scoring of eukaryotic, prokaryotic, and viral genomes. Mol Biol Evol.

[43] Mukherjee K., Campos H., Kolaczkowski B. (2013). Evolution of Animal and Plant Dicers: Early Parallel Duplications and Recurrent Adaptation of Antiviral RNA Binding in Plants. Mol Biol Evol.

[44] Zhao H. (2023). Species diversity, updated classification and divergence times of the phylum Mucoromycota. Fungal Divers.

[45] Jespersen N., Monrroy L., Barandun J., Weiss L.M., Reinke A.W. (2022). Experientia Supplementum.

[46] Qin S. (2023). Molecular characterization reveals no functional evidence for naturally occurring cross-kingdom RNA interference in the early stages of *Botrytis cinerea* –tomato interaction. Mol Plant Pathol.

[47] Kelani A.A. (2023). Disruption of the Aspergillus fumigatus RNA interference machinery alters the conidial transcriptome. RNA.

[48] Carreras-Villaseñor N., Esquivel-Naranjo E.U., Villalobos-Escobedo J.M., Abreu-Goodger C., Herrera-Estrella A. (2013). The RNAi machinery regulates growth and development in the filamentous fungus *T richoderma atroviride*. Mol Microbiol.

[49] Enriquez-Felix E.E. (2024). Argonaute and Dicer are essential for communication between *Trichoderma atroviride* and fungal hosts during mycoparasitism. Microbiol Spectr.

[50] Villalobos-Escobedo J.M. (2022). Trichoderma atroviride hyphal regeneration and conidiation depend on cell-signaling processes regulated by a microRNA-like RNA. Microb Genom.

[51] Lee H.-C. (2010). Diverse Pathways Generate MicroRNA-like RNAs and Dicer-Independent Small Interfering RNAs in Fungi. Mol Cell.

[52] Bailey T.L., Johnson J., Grant C.E., Noble W.S. (2015). The MEME Suite. Nucleic Acids Res.

[53] Yan Y., Zhang D., Zhou P., Li B., Huang S.-Y. (2017). HDOCK: a web server for protein–protein and protein–DNA/RNA docking based on a hybrid strategy. Nucleic Acids Res.

[54] Tian Y. (2014). A Phosphate-Binding Pocket within the Platform-PAZ-Connector Helix Cassette of Human Dicer. Mol Cell.

[55] Bernstein E. (2003). Dicer is essential for mouse development. Nat Genet.

[56] Gasciolli V., Mallory A.C., Bartel D.P., Vaucheret H. (2005). Partially Redundant Functions of Arabidopsis DICER-like Enzymes and a Role for DCL4 in Producing trans-Acting siRNAs. Curr Biol.

[57] Cánovas-Márquez J.T. (2021). A ribonuclease III involved in virulence of Mucorales fungi has evolved to cut exclusively single-stranded RNA. Nucleic Acids Res.

[58] Wei X. (2021). Structural basis of microRNA processing by Dicer-like 1. Nat Plants.

[59] A.J. Malik, C. Puente-Lelievre, N. Matzke, D.B. Ascher, On use of tertiary structure characters in hidden Markov models for protein fold prediction. [Preprint] (2024). Available at: 〈http://biorxiv.org/lookup/doi/10.1101/2024.04.08.588419〉 [Accessed 19 May 2025].

[60] Benavides T., Bystroff C. (May 2025). HMMSTRTM A hidden Markov Model Local Struct Predict Globul Membr Assoc Proteins [Prepr] (2023.

[61] Bystroff C., Thorsson V., Baker D. (2000). HMMSTR: a hidden Markov model for local sequence-structure correlations in proteins 1 1Edited by J. Thornton. J Mol Biol.

[62] Kans, J. Entrez Direct*:* E-utilities on the Unix Command Line. 2013 Apr 23.

[63] Raman V. (2017). Small RNA Functions Are Required for Growth and Development of *Magnaporthe oryzae*. MPMI.

[64] Potter S.C. (2018). HMMER web server: 2018 update. Nucleic Acids Res.

[65] Jones P. (2014). InterProScan 5: genome-scale protein function classification. Bioinformatics.

[66] Mirdita M. (2022). ColabFold: making protein folding accessible to all. Nat Methods.

[67] Dolinsky T.J., Nielsen J.E., McCammon J.A., Baker N.A. (2004). PDB2PQR: an automated pipeline for the setup of Poisson-Boltzmann electrostatics calculations. Nucleic Acids Res.

[68] Søndergaard C.R., Olsson M.H.M., Rostkowski M., Jensen J.H. (2011). Improved Treatment of Ligands and Coupling Effects in Empirical Calculation and Rationalization of p *K*_a_ Values. J Chem Theory Comput.

[69] Olsson M.H.M., Sondergaard C.R., Rostkowski M., Jensen J.H. (2011). PROPKA3: consistent treatment of internal and surface residues in empirical pKa predictions. J Chem Theory Comput.

[70] Baker N.A., Sept D., Joseph S., Holst M.J., McCammon J.A. (2001). Electrostatics of nanosystems: Application to microtubules and the ribosome. Proc Natl Acad Sci USA.

[71] Dong R., Pan S., Peng Z., Zhang Y., Yang J. (2018). mTM-align: a server for fast protein structure database search and multiple protein structure alignment. Nucleic Acids Res.

[72] Shannon P. (2003). Cytoscape: A Software Environment for Integrated Models of Biomolecular Interaction Networks. Genome Res.

[73] Notredame C., Higgins D.G., Heringa J. (2000). T-coffee: a novel method for fast and accurate multiple sequence alignment 1 1Edited by J. Thornton. J Mol Biol.

[74] Thompson J.D., Higgins D.G., Gibson T.J. (1994). CLUSTAL W: improving the sensitivity of progressive multiple sequence alignment through sequence weighting, position-specific gap penalties and weight matrix choice. Nucleic Acids Res.

[75] Steenwyk J.L., Buida T.J., Li Y., Shen X.-X., Rokas A. (2020). ClipKIT: A multiple sequence alignment trimming software for accurate phylogenomic inference. PLoS Biol.

[76] Minh B.Q. (2020). IQ-TREE 2: New Models and Efficient Methods for Phylogenetic Inference in the Genomic Era. Mol Biol Evol.

[77] Wong T.K., Kalyaanamoorthy S., Meusemann K., Yeates D.K., Misof B., Jermiin L.S. (2020). A minimum reporting standard for multiple sequence alignments. NAR Genom Bioinforma.

[78] Revell L.J. (2012). phytools: an R package for phylogenetic comparative biology (and other things). Methods Ecol Evol.

[79] Zhu Q., Kosoy M., Dittmar K. (2014). HGTector automated method facilitating genomewide discovery putative horizontal gene transfers.

[80] Buchfink B., Xie C., Huson D.H. (2015). Fast and sensitive protein alignment using DIAMOND. Nat Methods.

[81] Huang J., MacKerell A.D. (2013). CHARMM36 all-atom additive protein force field: Validation based on comparison to NMR data. J Comput Chem.

[82] Phillips J.C. (2020). Scalable molecular dynamics on CPU and GPU architectures with NAMD. J Chem Phys.

[83] Katoh K., Standley D.M. (2013). MAFFT Multiple Sequence Alignment Software Version 7: Improvements in Performance and Usability. Mol Biol Evol.

[84] Johnson N.R., Gonzalez-Toro F., Gomez B.B. (2025). Class-agnostic annotation of small RNAs balances sensitivity and specificity in diverse organisms. Comput Struct Biotechnol J.

[85] Martin M. (2011). Cutadapt removes adapter sequences from high-throughput sequencing reads. EMBnet. journal.

[86] Johnson N.R., Yeoh J.M., Coruh C., Axtell M.J. (2016). Improved Placement of Multi-mapping Small RNAs. G3 Genes Genomes Genetics.

[87] Grant B.J., Skjærven L., Yao X. (2021). The Bio3D packages for structural bioinformatics. Protein Sci.

