# Supplementary material for: Clade-wide fungal proteome analysis reveals structure–function conservation in divergent Dicer proteins
Source: Comput Struct Biotechnol J. 2025 Nov 6;27:5020–35. doi: 10.1016/j.csbj.2025.11.009 (PMC12663654; doi:10.1016/j.csbj.2025.11.009)
Supplement: Supplementary file 1 — Supplementary material [file mmc1.docx]

**Supplementary Figures**


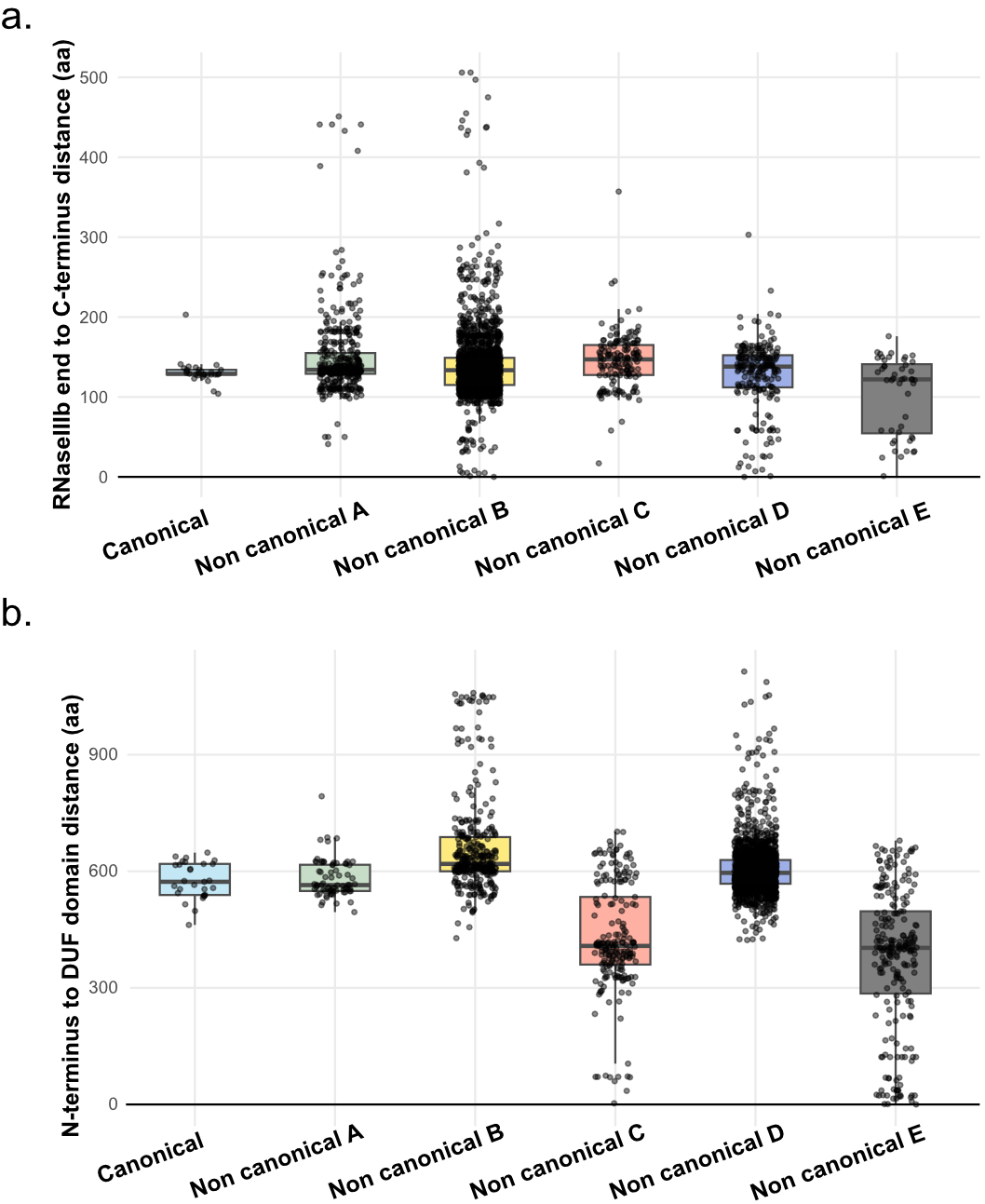


**Supplementary Figure 1.** **Domain spacing in canonical and non-canonical fungal Dicer proteins.** (**a**) Distance in amino acids from the RNaseIIIb domain to the C-terminus (**b**) Distance in amino acids from the N-terminus to the DUF domain. Each dot represents a single protein. Boxplots summarize median and interquartile ranges for canonical and non-canonical DCR categories.


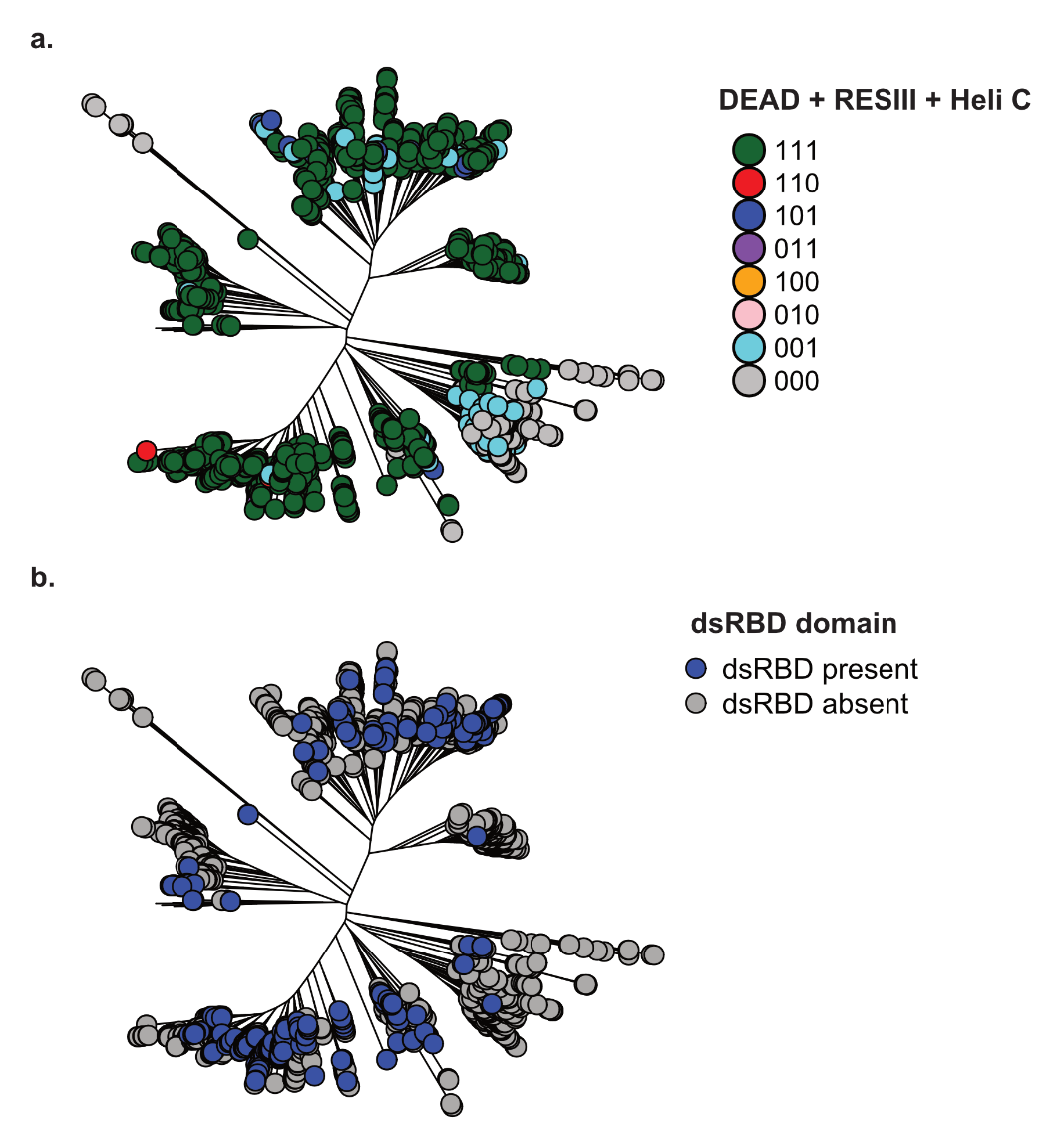


**Supplementary Figure 2.** **Helicase and dsRBD composition of fungal Dcr proteins mapped onto the phylogeny.** (**a**) Distribution of helicase domains subcomponents (DEAD, RESIII, and Helicase) across fungal Dcr proteins. Each tip in the phylogeny is colored on a binary triplet indicating the presence or absence of helicase subdomains in the order: DEAD, RESIII, and Helicase C (Heli_C). Green tips (111) indicate complete helicase domains, while grey tips (000) correspond to complete absence of these subdomains. (**b**) Presence or absence of the canonical dsRBD in fungal Dcr proteins. Presence (blue tips) or absence (no visible tip) of the canonical dsRNA-binding domain (dsRBD) across the same phylogenetic tree.


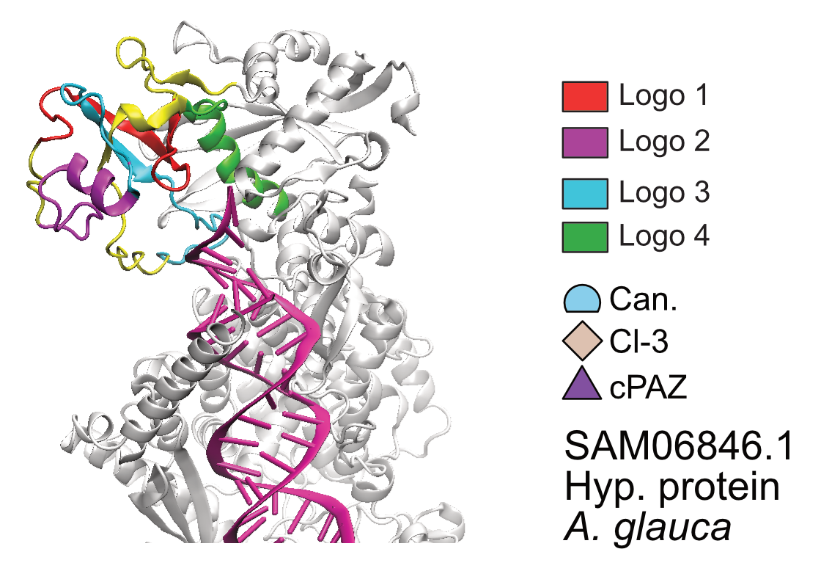


**Supplementary Figure 3.** **Spatial localization of conserved PAZ motifs in a canonical Dicer protein.** Mapping of sequence logos (Logos 1–4) derived from conserved motifs within the canonical PAZ domain onto the 3D structure of a Dicer protein from *Absidia glauca* (SAM06846.1), modeled in complex with a pre-cleaved dsRNA substrate that was obtained from the PDB model of *D. melanogaster* Dicer (pdb_00007w0f) (dsRNA, magenta). The protein backbone is shown in white. The core PAZ domain is highlighted in yellow, while each conserved motif is colored individually: Logo 1 (red), Logo 2 (purple), Logo 3 (cyan), and Logo 4 (green).

**
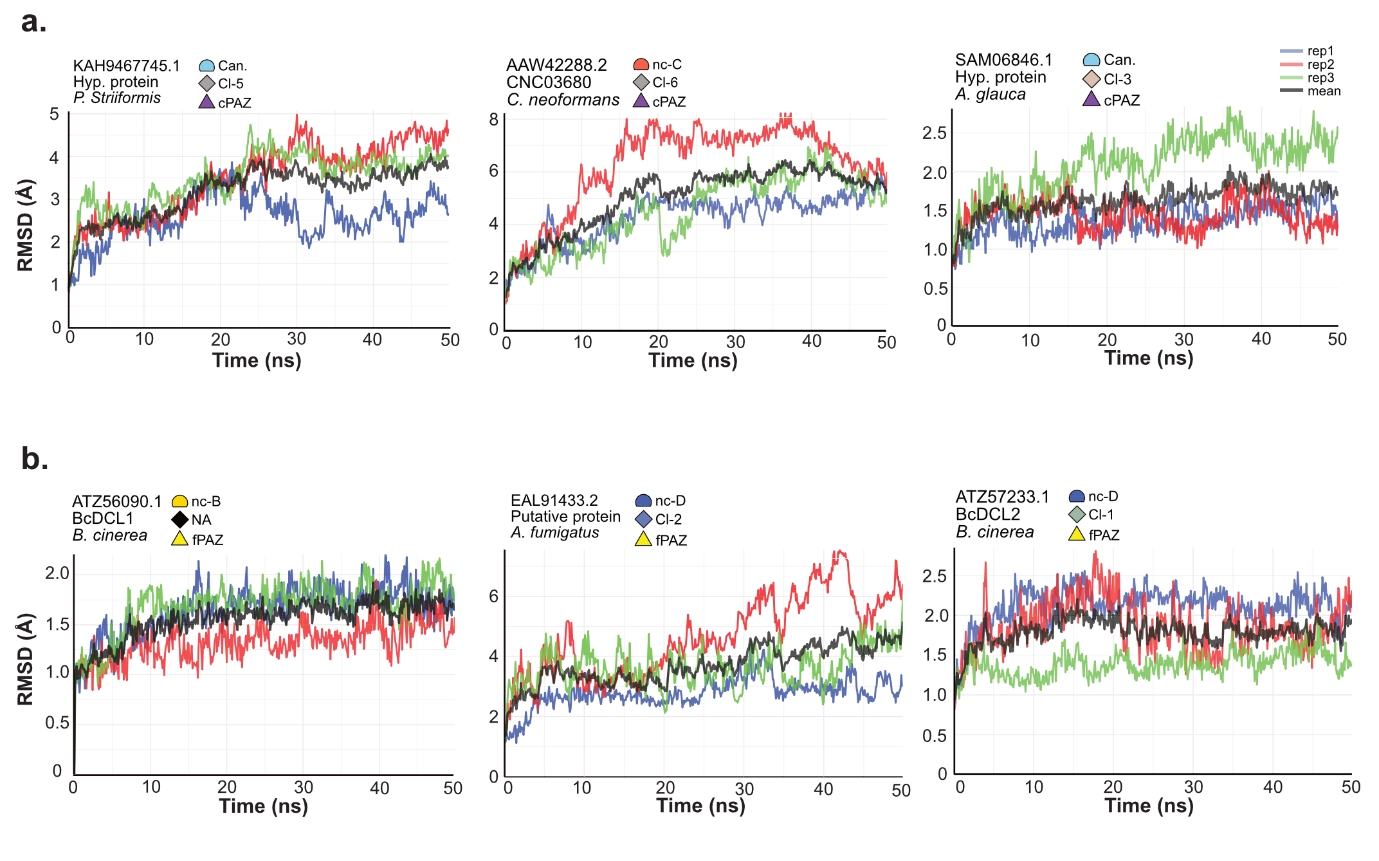
**

**Supplementary Figure 4.** **Structural stability of Dicer–RNA complexes during molecular dynamics simulations.** Root mean square deviation (RMSD, in Å) over a 50-nanosecond (ns) molecular dynamics trajectory for six representative Dicer–RNA complexes. (**a**) Dcr proteins containing a canonical PAZ domain (cPAZ), (**b**) Dcr proteins containing a fungal-specific PAZ domain (fPAZ), as defined in this study. Each plot shows the RMSD trajectories from three independent replicates, replicate 1 (blue), replicate 2 (red), and replicate 3 (green), with the black line indicating the mean RMSD across replicates.
